# Notch and PKC Are Involved in Formation of the Lateral Region of the Dorso-Ventral Axis in Drosophila Embryos

**DOI:** 10.1371/journal.pone.0067789

**Published:** 2013-07-04

**Authors:** Daniel M. Tremmel, Sedat Resad, Christopher J. Little, Cedric S. Wesley

**Affiliations:** Departments of Genetics and Medical Genetics, University of Wisconsin-Madison, Madison, Wisconsin, United States of America; University of Otago, New Zealand

## Abstract

The *Notch* gene encodes an evolutionarily conserved cell surface receptor that generates regulatory signals based on interactions between neighboring cells. In Drosophila embryos it is normally expressed at a low level due to strong negative regulation. When this negative regulation is abrogated neurogenesis in the ventral region is suppressed, the development of lateral epidermis is severely disrupted, and the dorsal aminoserosa is expanded. Of these phenotypes only the anti-neurogenic phenotype could be linked to excess canonical Notch signaling. The other phenotypes were linked to high levels of Notch protein expression at the surface of cells in the lateral regions indicating that a non-canonical Notch signaling activity normally functions in these regions. Results of our studies reported here provide evidence. They show that Notch activities are inextricably linked to that of Pkc98E, the homolog of mammalian PKCδ. Notch and Pkc98E up-regulate the levels of the phosphorylated form of IκBCactus, a negative regulator of Toll signaling, and Mothers against dpp (MAD), an effector of Dpp signaling. Our data suggest that in the lateral regions of the Drosophila embryos Notch activity, in conjunction with Pkc98E activity, is used to form the slopes of the opposing gradients of Toll and Dpp signaling that specify cell fates along the dorso-ventral axis.

## Introduction

Developmental function of Notch was discovered in Drosophila embryos more than 70 years ago [1]. Since then studies in animals from hydra to humans indicate that Notch functions are (1) evolutionarily conserved, (2) initiated by local cell-cell interactions, (3) involved in a diverse array of developmental processes, (4) intricately integrated with other basic developmental pathways, and (5) based on more than one signaling mechanism. Notch function in the Central Nervous System (CNS) development in Drosophila embryos exemplifies the best-understood signaling mechanism. Clusters of 12–20 neuroectodermal cells in the ventral region of stage 8–9 embryos first acquire the potential to become neuronal cells by expressing genes of the Achaete Scute Complex (e.g., *achaete*). When Notch expressed on one proneural cell binds its ligand Delta expressed on a neighboring proneural cell, Notch is first cleaved in the extracellular region by the Kuzbanian/ADAM 10 protease and subsequently in the transmembrane region by the Presenilin/γ-Sectretase protease complex to release the Notch intracellular domain (N^intra^/NICD). N^intra^/NICD translocates to the nucleus and in association with the DNA binding protein Suppressor of Hairless (RBP-Jκ) promotes transcription of target genes. This signaling (referred to as canonical Notch signaling) is blocked in a few cells within each proneural cluster, which commit to the neuronal fate by expressing neuronal genes (e.g., *hunchback*) and differentiate the CNS. Canonical Notch signaling is activated in the remaining cells of proneural clusters, which commit to the alternate epidermal fate by expressing epidermal genes of the Enhancer of split Complex (*E(spl)C*, HES) [2]–[27].

Notch is also known to signal by other (non-canonical), poorly understood mechanisms in developmental events involving F-actin dynamics, the cytoskeleton, or the extracellular matrix [3], [29]–[34]. We recently reported a Notch activity that is not based on canonical Notch signaling [35]. This Notch activity becomes apparent when endogenous Notch activity is globally increased due to abrogation of negative regulation of *Notch* mRNA 3′ processing, by either a mutation in the *Notch* gene (*N^nd1^*) or in the Drosophila Polypyrimidine Tract Binding (dmPTB) protein gene *hephaestus (heph^03429^)*. In the ventral region of mid-to-late stage *N^nd1^* and *heph^03429^* embryos (after stage 9) Notch protein depletes in conjunction with excess canonical Notch signaling and suppression of neurogenesis. In the lateral regions of the same embryos, Notch protein accumulates at the cell surface in conjunction with very high levels of F-actin and disruption of many processes that depend on proper development of the lateral epidermis (including dorsal closure and cardiogenesis). And in the dorsal region the extra-embryonic tissue amnioserosa is hyperplasic. Over-production of canonical Notch signaling through expression of N^intra^/NICD transgene or the classical *Notch* mutant *l(1)N^B^* allele reproduces the anti-neurogenic phenotype in the ventral region but not any of the phenotypes in the lateral or dorsal regions [35]. Since reproduction of phenotypes by N^intra^/NICD expression is proof of the involvement of canonical Notch signaling, our data suggested that this signaling is not involved in the phenotypes observed in the lateral and dorsal regions of *N^nd1^* and *heph^03429^* embryos.

The mutant phenotypes in the lateral and dorsal regions of *N^nd1^* and *heph^03429^* embryos are not consequences of non-physiological levels of Notch expression in the lateral regions (i.e., they are not neomorphs) because they are not observed in Delta null embryos or Suppressor of Hairless over-expressing embryos that also accumulate Notch protein well above physiological levels [2], [8], [12]. In other words, the presence of Delta or the loss of Suppressor of Hairless appears to be required in addition to Notch accumulation for the mutant phenotypes to develop, which implicates Notch activity as the underlying factor (not mere Notch over-expression). Several lines of evidence [35] indicate that the mutant phenotypes in *N^nd1^* and *heph^03429^* embryos are related to functions that Notch normally performs in the lateral regions. For example, the patterns of actin over-expression in mutant embryos correspond to the patterns of high actin expression in the lateral epidermis of wild type embryos. Another example is the production of excess pericardial cells that is expected from excess Notch activity in the lateral region. Compelling evidence is also provided by experiments using Drosophila cultured cells. Schneider 2 (S2) cells express neither Notch nor Delta but can be made to express one or the other protein using transgenes. S2-Notch cells treated with S2-Delta cells recapitulate all known aspects of Notch function [5], [6], [8], [9], [12], [16], [19], [25]–[27], [35]. Experiments with these cells show that F-actin accumulates near the Notch receptor clusters that form at S2-Notch cell surfaces in contact with S2-Delta cells. This accumulation subsides over time as the level of Notch at the cell surface decreases and the level of N^intra^/NICD increases. Clone 8 is another Drosophila cell line. It expresses Notch from the endogenous gene but not Delta. When these cells are incubated with S2-Delta cells for prolonged periods cell fusions are observed suggesting that Notch and Delta interaction might underlie cell fusions observed in the lateral regions of *N^nd1^* and *heph^03429^* embryos [35]. Thus, our published data suggested that a Notch function is required in the lateral regions of the embryos and that this function might involve signaling activity at the cell surface (in addition to ligand binding) rather than canonical Notch signaling activity in the nucleus.

Our efforts to understand Notch function in the lateral regions led to the discovery of Notch function in dorso-ventral (dv) axis formation reported here. Formation of the dv axis is one of the early developmental events in Drosophila embryogenesis. It takes place in the mono-layered epithelium that occupies the periphery of the embryo (at stage 5). One of the primary factors involved is the Toll receptor. It is activated in a ventral (high) to dorsal (low) gradient that results in a corresponding gradient of degradation of Cactus (the Drosophila homolog of IκB) and release of Dorsal (the Drosophila homolog of NFκB) from its cytoplasmic tether. Released Dorsal translocates to the nucleus, becoming phosphorylated in the process, and turns on the expression of genes responsible for specifying the more ventral cell fates. The other important products in cell fate specification along the dv axis relate to the activity of the morphogen Decapentaplegic (Dpp), the Drosophila homolog of Bone Morphogenetic Protein (BMP). Dpp is secreted in the dorsal region either in an inactive form or is kept inactive in the extracellular space/matrix by inactivating proteins. Upon activation (by proteases) DPP binds its receptor resulting in phosphorylation of the Mothers against dpp (MAD) transcription factor. MAD translocates to the nucleus and activates genes specifying the more dorsal fates. Cells in the lateral regions of the embryo acquire different fates by activating different sets of genes according to their position along the opposing gradients of Toll and Dpp/BMP signaling. The ventral-most cells become the mesodermal cells, which are followed by a narrow band of mesectodermal cells that define the dorsal limits of the mesodermal cell specification. The next broad band of cells is composed of the neuroectodermal cells from which the CNS later differentiates. These cells are followed by cells that differentiate the lateral epidermis and the very specialized band of cells called the leading edge cells that regulate the dorsal closure process and the differentiation of organs such as the cardio-vascular system. The dorsal most cells form the amnioserosa, which contributes to the dorsal closure process and differentiation of organs from the lateral epidermis [36]–[49].

Maternal factors that are spatially arranged in the embryo and perivitelline fluid are thought to activate different genes at different positions along the dv axis. In other words, the mother is thought to predetermine the information for dv axis formation and the newly formed cells are thought to merely respond to this information without generating new information. One way cells generate new information is via inter-cellular interactions mediated by Notch. Canonical Notch signaling is activated in the narrow band of mesectodermal cells in between the ventral mesodermal cells and the lateral neuroectodermal cells. It is not known whether this activation is completely pre-determined by maternal factors or includes new information generated by interaction between neighboring, equipotent cells. Canonical Notch signaling is blocked in the mesodermal cells by the transcription repressor Snail. The mechanism includes suppression of Delta expression but is more complex because it silences even N^intra^/NICD expressed from a transgene, which does not require Delta for activity. Canonical Notch signaling is also blocked in the neuroectodermal cells at this stage by an unknown mechanism that appears to suppress N^intra^/NICD production since transgenic expression of N^intra^/NICD overcomes this block [37], [50]–[52]. The data reported here might be related to the blocking mechanism in the lateral neuroectodermal cells but that would be incidental. The main implication is that a non-canonical Notch activity is involved in forming the slopes of the opposing gradients of Toll and Dpp signaling in the lateral regions of the Drosophila embryo. This Notch activity involves the activity of Protein Kinase C (PKC), a kinase that requires plasma membrane association for activation and is known to regulate a wide array of processes including those regulated by calcium and cyclicAMP. Notch and PKC activities are in a regulatory loop that makes their empirical dissection difficult but present a great opportunity for understanding how events at the cell surface and nucleus are integrated.

## Results

### Notch Promotes Expression of Phosphorylated Cactus in Drosophila Embryos

**Figure 1:**
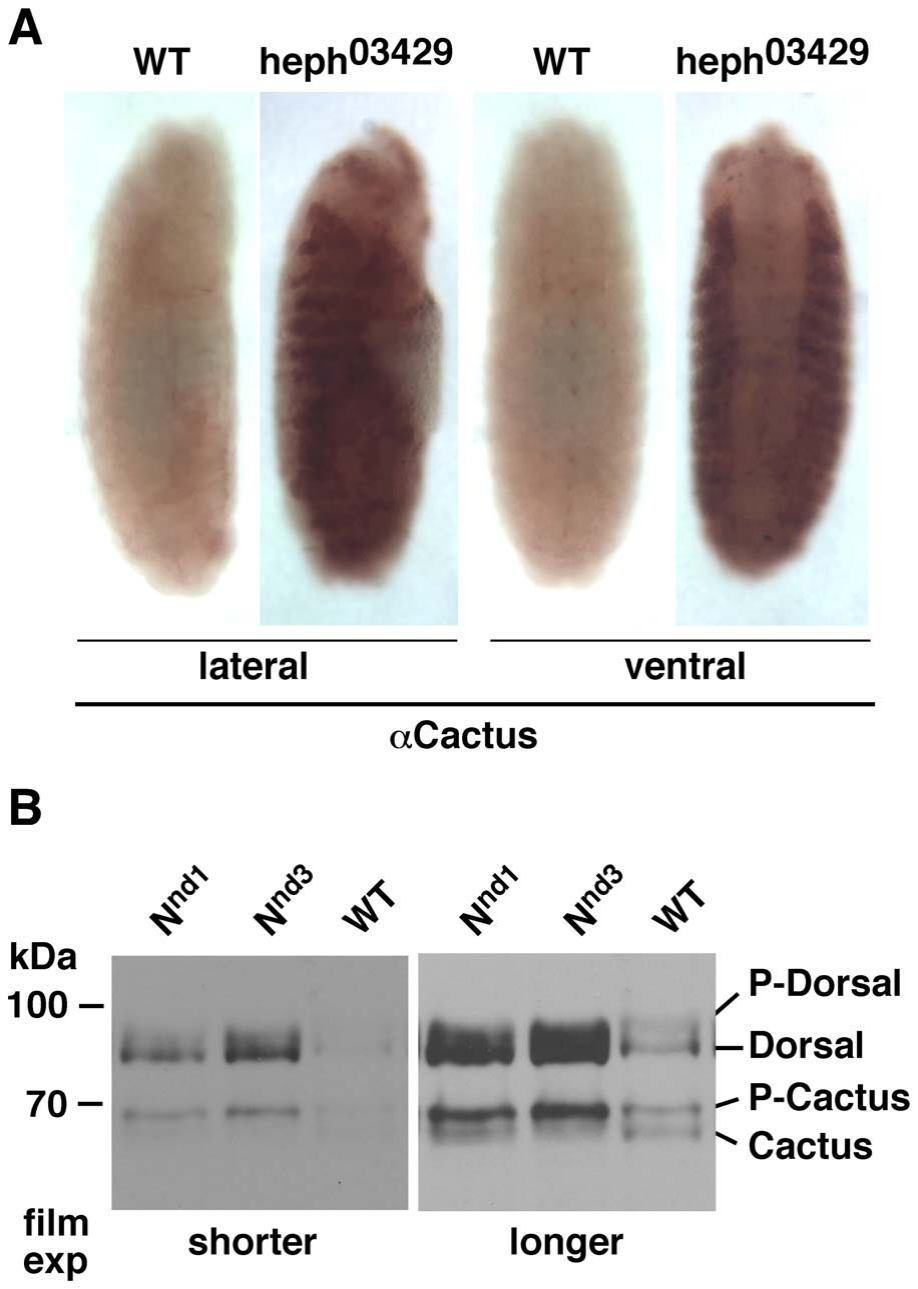
P-Cactus and P-Dorsal levels in embryos that over-express Notch globally. **A**. Cactus accumulates to a high level in the lateral regions of *heph^03429^* embryos. Wild type (wt) strain used here and throughout the study is the *yellow white* (*yw*) strain. Embryos shown are at stages 13–14 probed with the Cactus antibody. All staging in this study was done according to [49]. **B**. *N^nd1^* and *N^nd3^* embryos express higher levels of P-Cactus in association with higher levels of Dorsal (the unphosphorylated, cytoplasmic form) [43]–[46]. Two different exposures to film are shown for clarity. The same blot was probed with the two different antibodies. Eggs of all genotypes were collected at room temperature at 1–3 hour intervals and incubated at 30°C for 1–3 hours before protein extraction. The same result was obtained from all samples. Data for 3-hour collection and 1-hour 30°C incubation are shown. The same number of embryos was used in each extraction and the same amount of the sample was loaded in each lane. Immuno-labeling experiments were repeated twice and most embryos of each stage showed the same phenotype. Western blotting experiments were repeated three times.

Immuno-labeling experiments first showed that Cactus expression was greatly increased in the lateral regions of embryos manifesting gain of function Notch phenotypes. Data from *heph^03429^* embryos are shown in **Figure 1A**. We next performed western blotting analysis. For this purpose we extracted total proteins instantly in the Laemmli gel loading buffer, as it most accurately represents Notch level at the time of extraction and recovers proteins from all sub-cellular compartments. As total proteins in Laemmli buffer cannot be quantified accurately, we resorted to using the same number of embryos for the samples being compared and loaded the same amount of these samples onto gels. This method results in an even loading of total proteins (please see the Hsp70 panel in **Figure S1**). In many of our blots the differential accumulation of the different proteins within and between lanes itself provides support to the conclusions we draw. Where necessary, we use background bands or show Hsp70 levels to support our interpretation.

Western blotting analysis of gain of function *Notch* embryos is shown in **Figure 1B**. As is apparent, gain in Notch activity results in accumulation of the phosphorylated form of Cactus (which we will refer to as P-Cactus) in association with a higher level of the unphosphorylated form of Dorsal. We observe that same effect in *heph^03429^* embryos (see later). The unphosphorylated form of Dorsal is the cytoplasmic form and the phosphorylated form (which we will refer to as P-Dorsal) is the nuclear form [46]. Our studies confirm that P-Cactus is cytoplasmic and P-Dorsal is nuclear (see later). *N^nd3^* is another temperature sensitive *Notch* allele with a mis-sense mutation in the amino terminus of the coding region that results in gain of Notch signaling phenotypes at the restrictive temperature of 30°C, similar to the *N^nd1^* allele [16], [21]. Both *N^nd1^* and *N^nd3^* embryos over-produce the Notch protein (**Fig. S1**). Western blotting analysis of embryos at different stages indicates that the effect of Notch on P-Cactus can be observed even in stage 16 or older embryos in which Dorsal is almost undetectable.

### Notch Promotes Expression of P-Cactus and Dorsal in Drosophila Cultured Cells

**Figure 2:**
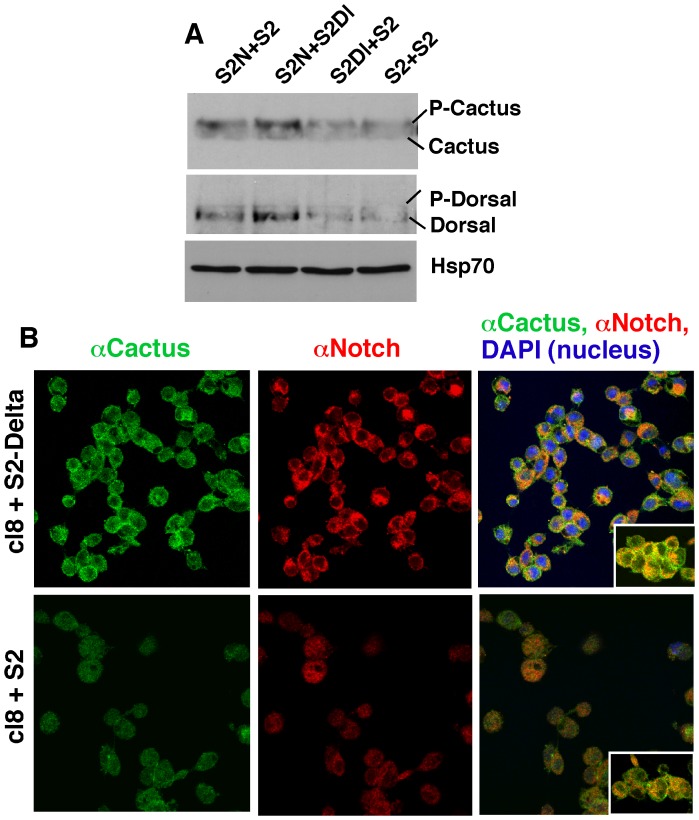
Effect of Notch activation on Cactus and Dorsal in Drosophila cultured cells. **A**. Notch activation in S2 cells increases the level of P-Cactus and Dorsal. S2N = S2-Notch cells, S2Dl = S2-Delta cells, S2 = S2 cells expressing neither Notch nor Delta. Equal numbers of the different cell types were mixed and aggregated for 20 minutes at room temperature before protein extraction. The same number of cells was used for each aggregation sample. Total proteins were extracted with the Laemmli buffer and the same amount of extract was used in each lane. The level of housekeeping protein Hsp70 shows that the same amount of total protein is loaded in each lane. **B.** Notch activation in clone 8 (cl8) cells results in transient accumulation of Notch and Cactus at the cell surface. cl8 cells express Notch endogenously. Insets show extreme instances of cell surface Notch and Cactus localization that we have observed in the two treatments. Highly cross-adsorbed secondary antibodies were used to suppress cross-reactivity. Both western blotting and immuno-labeling experiments were repeated three times.

*N^nd1^* embryos incubated at 30°C for just 30 minutes (at any stage) accumulated P-Cactus indicating that Notch activity and P-Cactus level are closely linked. We tested it in experiments with S2 cells. As mentioned earlier, these cells express neither Notch nor Delta but can be made to express them from transgenes. By comparing the response between cells with or without Notch (i.e., S2-Notch and S2 cells, respectively) and between cells in which Notch is activated (S2-Notch+S2-Delta) or not (S2-Notch+S2) we can determine whether Notch activation directly affects P-Cactus level. Results of our experiments showed that the levels of P-cactus and Dorsal were higher in S2-Notch cells treated with S2-Delta cells (**Fig. 2A**). We also found that the impact of Notch activation on P-Cactus and Dorsal levels was observed only within the first 30 minutes. Clone 8 cell line (cl8) is another commonly used Drosophila cultured cells. These cells express Notch endogenously but not Delta. When treated with S2-Delta cells, cl8 cells reproduce all known aspects of Notch activation [12], [54]. cl8 cells treated with S2-Delta affected the expression of P-Cactus and Dorsal in the same manner as S2-Notch cells indicating that these effects were not linked to heat shock induction (used for Notch expression in S2-Notch cells) or the use of a transgenic system. We next examined whether Notch activation impacted subcellular distribution of Cactus and found that both Notch and Cactus enriched at the cell surface soon after cl8 and S2-Delta cells were mixed (**Fig. 2B**). Cactus enrichment at the cell surface significantly reduced after 30 minutes of Delta treatment, consistent with results from S2 cells.

The response of P-Cactus and Dorsal to Notch activation was comparable to that of F-actin that accumulates soon after S2-Notch and S2-Delta are mixed and then subsides in conjunction with loss of Notch from the cell surface [35]. Since it is well known that *E(spl)C m3* mRNA, the target of canonical Notch signaling in S2 cells, takes about 45 minutes for peak expression after S2-Notch and S2-Delta cells are mixed [12], [25], our P-Cactus and F-actin data were suggesting that Notch and Delta affected the expression of a different set of genes soon after they bind. We confirmed it with experiments in S2 cells that enable rigorous controls. *ovo-shavenbaby* (*ovo-svb*) is a master regulator of actin dynamics [55]–[57] and our microarray analysis had shown that its mRNA responds to Notch activation (we did not find evidence of response by *cactus* or *dorsal* mRNA). Results of time course experiments showed that while *ovo-svb* mRNA level peaked soon after mixing S2-Notch cells and S2-Delta cells, i.e., at 0 min incubation, the level of *E(spl)C m3* mRNA was low at that time and peaked 45 minutes later, at which time the level of *ovo-svb* had subsided to the background level (**Fig. S2A**). Note that the same blot was probed for the expression of different genes. Western blotting analysis of samples from the same cell populations showed that N^intra^/NICD level was at the background level soon after mixing and accumulated to a high level at 45 minutes (**Fig. S2B**). Thus, Notch exhibits one activity soon after Delta binding, when it is stabilized at the cell surface, and a different (canonical) activity later on, when it is cleaved to produce N^intra^/NICD. Our immuno-labeling data from *N^nd1^* and *heph^03429^* embryos together with time course and sub-cellular re-distribution data from cultured cells suggest that the responses of P-Cactus and Dorsal proteins are linked to Notch activity at the cell surface rather than in the nucleus.

### Loss of Notch Activity in Embryos has the Opposite Effect on P-Cactus and Dorsal

**Figure 3:**
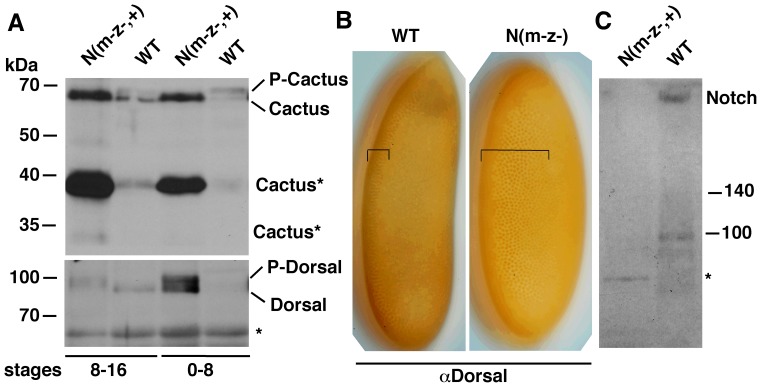
Loss of Notch expression affects P-cactus and P-Dorsal levels as well as the extent of nuclear localization of Dorsal along the dv axis. **A**. Notch null embryos express low levels of P-Cactus and high levels of P-Dorsal. N(m-z-,+) = Embryos composed of 50% maternal null/zygotic Notch nulls and 50% maternal Notch null/zygotic wild type. Cactus* = putative degradation/cleaved Cactus forms. Extracts from stage 8–16 embryos (first two lanes) and stage 0–8 embryos (last two lanes) are shown. **B**. Nuclear Dorsal extends deep into the lateral regions in maternal and zygotic Notch null embryos (N(m-z-)) embryos. **C**. Notch expression is reduced in N(m-z-,+) embryos. Protein extracts from stage 0–16 embryos were used. The two blots in **A** were generated using the same samples. The same number of embryos was used in each lane in **A** and **C**. * = a non-specific band that serves to also indicate protein loading is not an issue. The western blotting experiments were repeated seven times and the immuno-labeling experiments twice. In the latter, most embryos at a particular stage showed the same phenotype.

**Figure 4:**
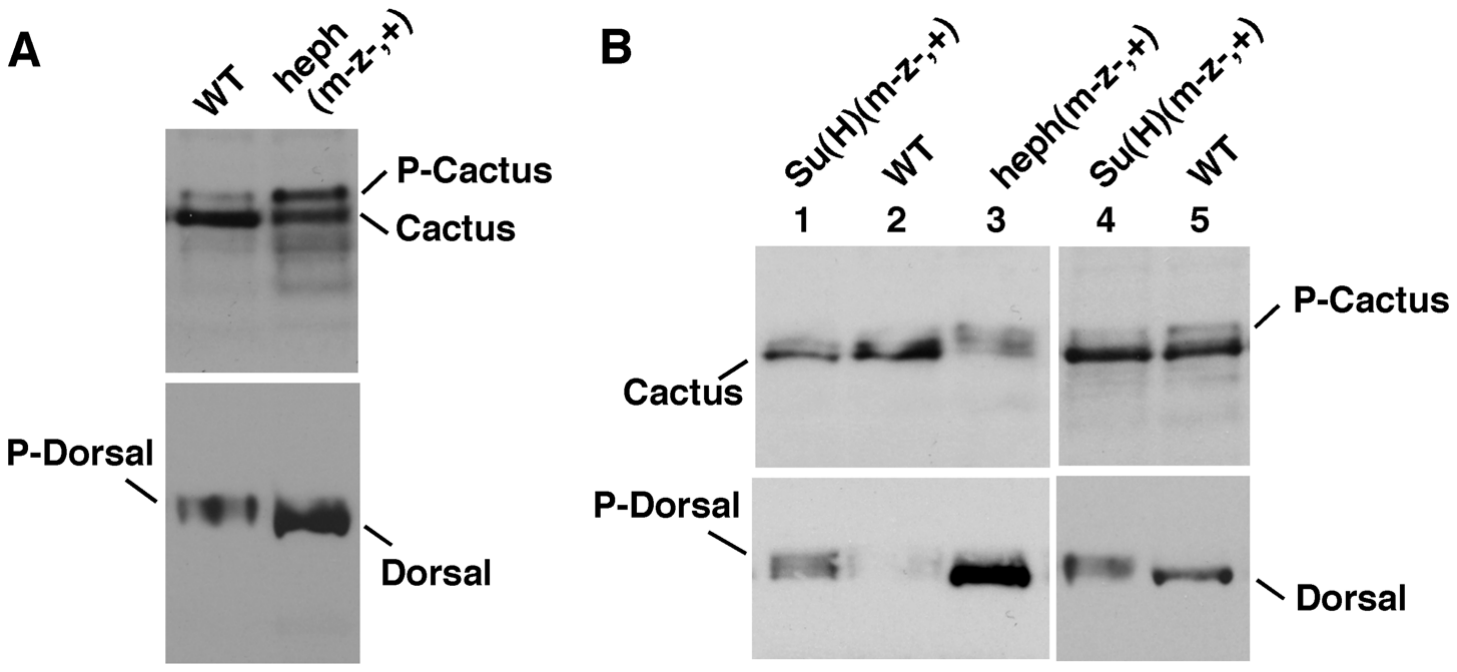
Gain and loss of Notch activity affects P-Cactus and Dorsal levels in an opposite manner. **A**. Embryos manifesting gain of Notch activity, maternal and zygotic *heph^03429^* null embryos (heph(m-z-,+)), express a high level of P-Cactus and a low level of P-Dorsal. **B**. Embryos manifesting loss of Notch activity, maternal and zygotic Suppressor of Hairless null embryos (Su(H)(m-z-,+) embryos, express a low level of P-Cactus and a higher level of P-Dorsal. Extracts from stage 0–8 embryos (lanes 1–3) or stage 0–6 (lanes 4–5) are shown. The two blots in **A** and **B** were generated using the same samples. The same number of embryos was used and the same amount of the extract was loaded in each lane of a blot. heph(m-z-,+) experiments were repeated 8 times and Su(H)(m-z-,+) experiments four times.

If Notch regulation of P-Cactus level were a true function of the *Notch* gene, loss of Notch expression was predicted to show the opposite effect: reduction in P-Cactus and increase in P-Dorsal. We tested this prediction by studying maternal and zygotic *Notch* null embryos. For western blotting analyses we used populations that were composed of 50% maternal and zygotic nulls and 50% maternal null and zygotic wild type embryos (N(m^−^z^−,+^)); for immuno-labeling analyses we picked embryos that were maternal and zygotic nulls (N(m^−^z^−^)). We obtained three very significant results. First, Cactus (not P-Cactus) accumulates in N(m^−^z^−,+^) embryos in conjunction with high levels of P-Dorsal (**Fig. 3A**). Second, a large amount of a truncated form of Cactus accumulates in N(m^−^z^−,+^) embryos (Cactus* in **Fig. 3A**). Third, the ventral band of nuclear Dorsal expands into the lateral regions of N(m^−^z^−^) embryos as could be predicted from the higher level of P-Dorsal on western blots (**Fig. 3B**). **Figure 3C** shows that the level of Notch was indeed reduced in N(m^−^z^−,+^) embryos. We further verified these results by studying maternal and zygotic nulls for *hephaestus*, the negative regulator of Notch activity. These heph(m^−^z^−,+^) embryos, which manifest gain of Notch activities, were expected to show the opposite effect: accumulate P-Cactus in association with the accumulation of Dorsal. **Figure 4A** shows that that is exactly what we find. We did yet another test to be sure we were on the right track. Loss of Suppressor Hairless eliminates not only the canonical nuclear Notch (N^intra^) function but also any other Notch function, as it is required for the stability of the full length Notch receptor [12]. The prediction here was that *Suppressor of Hairless* null embryos (Su(H) (m^−^z^−+^)), would express less P-Cactus and more nuclear Dorsal. Note that this is the opposite of what we predicted for the gain of Notch function heph(m^−^z^−,+^) embryos. Our results verify this prediction as well **(** **Fig. 4B**). We interpret the opposite effects of loss and gain of Notch activity to mean that Notch activity regulates the levels of P-Cactus and Dorsal.

### Gain of Notch Activity in the Lateral Regions Promote Dpp/BMP Signaling

**Figure 5:**
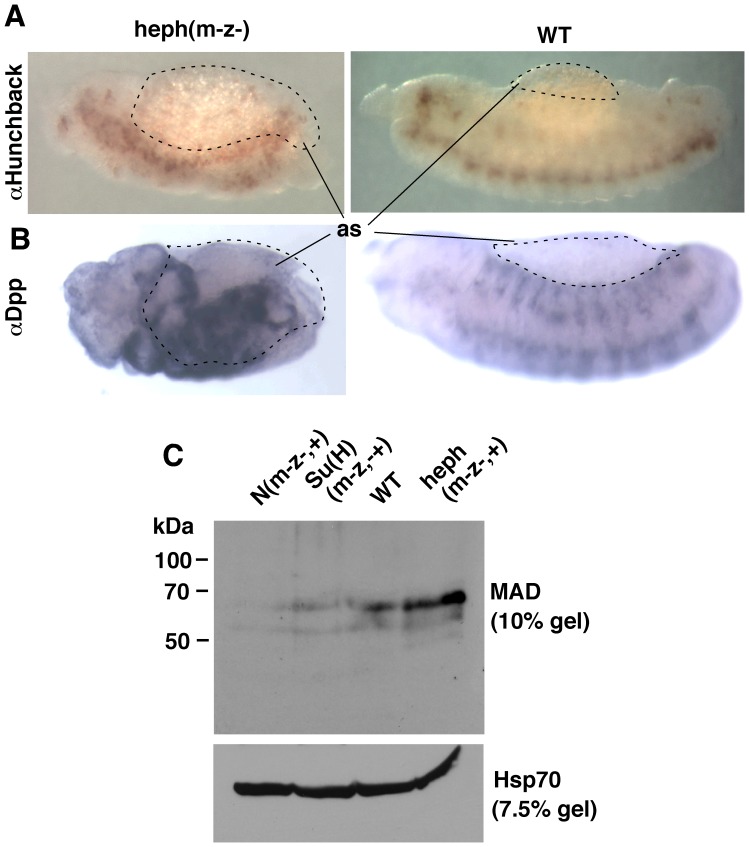
Gain and loss of Notch activity affects Dpp signaling in an opposite manner. **A**. In heph(m-z-) embryos that manifest gain of Notch activity, the dorsal amnioserosa expands at the expense of the lateral regions. **B**. Expression of Dpp is up regulated in heph(m-z-) embryos. **C**. While embryos that manifest loss of Notch activity (N(m-z-,+), SuH(m-z-,+)) express lower levels of MAD, embryos that manifest gain of Notch activity (heph(m-z-,+)) express a higher level of MAD. Extracts from stage 0–8 embryos are shown. The same number of embryos was used for protein extraction and the same amount of the extract was loaded in each lane of a blot. The same samples were separated in 10% and 7.5% percentage gels for probing with MAD and Hsp 70 antibodies. Immuno-labeling experiments were repeated two times and most embryos at a particular stage showed the same ohenotype. The western blotting experiments wre repeated three times.

Our data thus far had shown that Notch activity increases the levels of P-Cactus and cytoplasmic Dorsal concomitant with reduction in the level of nuclear P-Dorsal. As could be expected from these relationships, the ventral band of cells with nuclear Dorsal expanded into the lateral regions in N(m^−^z^−^) embryos. We wanted to find out whether the converse is also true: gain of Notch activity increases the expanse of the lateral region at the expense of the ventral region. For this purpose, we studied heph(m^−^z^−^) embryos that due to gain in Notch activity show high levels of P-Cactus and Dorsal and a low level of P-Dorsal. What we observed in these embryos was both unexpected and surprising. The lateral regions were almost absent in heph(m^−^z^−^) embryos and the amnioserosa was greatly expanded (**Fig. 5A**). The extent of the ventral region appeared to be unaffected and manifested loss of neuronal cells, which is consistent with the gain of canonical Notch signaling in this region. Thus, it appeared that in heph(m^−^z^−^)embryos the dorsal region expanded at the expense of the lateral regions (instead of the lateral regions expanding at the expense of the ventral region as we expected). To confirm that the dorsal region had expanded, we probed the embryos with an antibody against Dpp, the factor responsible for specifying dorsal fates. We observed high levels of Dpp in heph(m^−^z^−^) embryos (**Fig. 5B**). To determine whether it was just Dpp expression or Dpp signaling activity that was affected by Notch, we examined the level of MAD, the effector of Dpp signaling. We found a high level of MAD expression in heph(m^−^z^−,+^) embryos and low levels in SuH(m^−^z^−,+^) and N(m^−^z^−,+^) embryos (**Fig. 5C**). *N^nd1^* embryos also expressed a higher level of Dpp and MAD compared to the level in wild type embryos and manifested phenotypes similar to those in heph(m^−^z^−^) embryos. However the phenotypes exhibited lower expressivity and variable penetrance, which we attribute to the 30°C incubation time required for the temperature sensitive *N^nd1^* allele to manifest its effects. A significant number of embryos would have passed the dv axis formation stage by the time the *N^nd1^* allele assumes mutant status. We did not incubate *N^nd1^* females at the restrictive temperature (30°C) as it is well known that Notch function is required for oogenesis. Results from these studies suggest that Notch activity related to the regulation of P-Cactus and Dorsal impacts Dpp/BMP signaling, expanding the range of this signaling from the dorsal region into the lateral regions. Nuclear localization of Dorsal in the ventral most region persisted in heph(m^−^z^−^) embryos indicating that the Notch effect on P-Dorsal is limited to the lateral regions.

### Pkc98E is a Potential Factor Involved in Notch Activity Related to P-Cactus Expression

In parallel studies, on the role of Notch in long-term memory formation in adult flies, we had observed almost identical effects of Notch on cyclicAMP Response Element Binding Protein (CREB, product of the *CrebB-17A* gene): Notch accumulation at the cell surface promoted CREB phosphorylation. Thus, data from different projects indicated the involvement of a kinase activity with Notch accumulation at the cell surface. Both Cactus and CREB contain target sequences for many kinases but these sequences are very similar (i.e., they cannot point to a specific kinase with certainty). To identify the kinase involved, we initially took a bioinformatics approach mining the large amount of actual and predicted genetic and protein interaction data available for Drosophila genes (at NCBI, FlyBase, and UCSC Genome Browser websites). We explored the question ‘is there a Drosophila kinase that could interact with Notch, Cactus, CREB and F-actin?’ We found only one kinase, *Pkc98E*. We next examined whether these five proteins shared sequences as could be expected if they interacted with the same signaling complex. We knew that Notch and Cactus contain ankyrin repeats that could bind F-actin. Thus, our test proteins were Pkc98E and CREB since there is no information linking them physically to Notch or Cactus or actin. Remarkably, all five proteins share domains mapping to the ankyrin repeats of Notch (**Fig. S3**).

There are three PKC genes in Drosophila (not counting the atypical PKC) [58]–[60]. Only one of them *Pkc98E* is expressed in embryos and cultured cells (our wet lab data and data from genome-wide expression studies available at the FlyBase and UCSC Genome Browser websites). There is essentially no published information on this gene, possibly because a mutant and RNAi stocks have only now become available (thanks to the genome-wide gene disruption and RNAi projects). *Pkc98E* is the homolog of mammalian PKCδ (one of the genes belonging to the Novel class of PKC). Mammalian PKC genes play important roles in cell growth, differentiation, apoptosis, motility, secretion, and host defense. They are also implicated in many diseases, including cardiovascular diseases, cancer, and Alzheimer’s [61]–[66]. At the molecular level, mammalian PKC functions have been linked to Toll/NFκB and γ-secretase activities. The significance of the connection to Toll/NFκB signaling to our studies is obvious. α-secretase family of proteases includes ADAM10, the homolog of Kuzbanian that cleaves Notch in the extracellular domain and generates the substrate for Presenilin/γ-Sectretase complex for producing N^intra^/NICD (i.e., canonical Notch signaling in the nucleus). Thus, all available information indicated that PKC and Notch function in similar contexts. Therefore, we studied *Pkc98E* with respect to Notch activity related to P-Cactus and Dorsal in Drosophila embryos and cultured cells.

**Figure 6:**
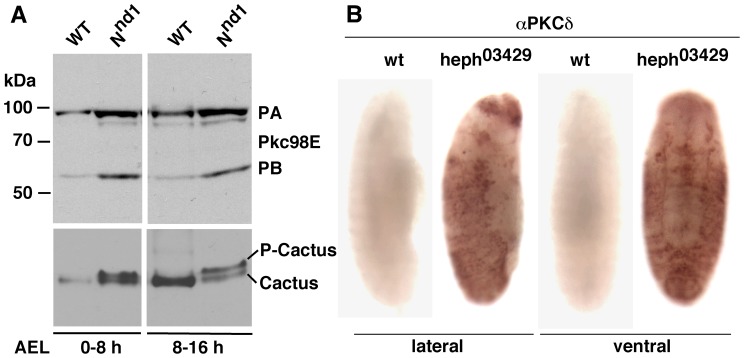
Notch activity promotes the expression of Pkc98E proteins in embryos. **A**. Notch gain of function *N^nd1^* embryos express higher levels of both the predicted forms of Pkc98E (PA and PB). The same blots probed for Cactus reveal that a high level of Pkc98E is associated with high levels of P-Cactus. The same number of embryos was used for all samples and the same amount of extract was loaded in each lane. **B**. *heph^03429^* embryos with gain in Notch activity express high levels of PKCδ in the lateral regions. Embryos shown are ∼ stage 14. Both the western blotting and the immuno-labeling experiments were repeated twice.

**Figure 7:**
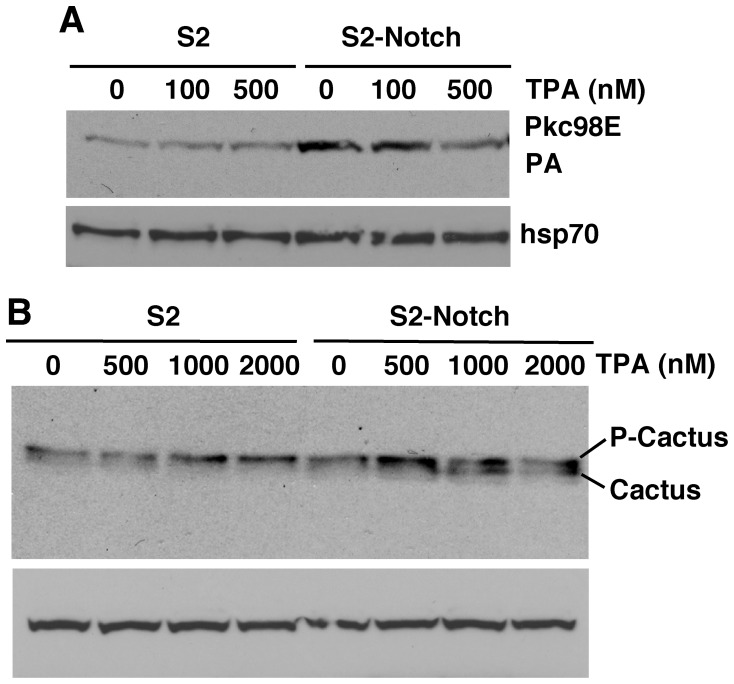
Notch potentiates PKC activity in S2 cells. **A**. S2-Notch cells express higher levels of Pkc98E. The high level in S2-Notch cells is reduced at high concentrations of the PKC activating drug phorbol ester (12-tetradecanoyl phorbol-13 acetate, TPA), which is expected because a high level of PKC activation is known to automatically trigger its down-regulation. **B**. P-Cactus level in S2-Notch cells increased at a lower concentration of TPA but decreased at high concentrations. The decrease was expected due to negative regulation of PKC at high concentrations of TPA. The same number of cells was used for each sample and the same amount of the sample was loaded in lane of the blot. The Pkc98E experiment was repeated twice and the Cactus/Dorsal experiment was repeated three times.

Since activation of a pathway alters the level of its primary components (due to turn-over or signaling related feedback regulation), we first examined the level of Pkc98E proteins in Notch gain of function embryos. We found that a high level of Pkc98E protein forms in conjunction with high levels of P-Cactus (**Fig. 6A**). Immuno-labeling studies showed that Pkc98E accumulates primarily in the lateral regions of embryos manifesting gain in Notch activity (**Fig. 6B**; data for *heph^03429^* is shown). Note that the lateral regions of *heph^03429^* embryos also accumulate Cactus (see Fig. 1) and Notch [51]. We next studied Pkc98E in cultured cells. We found that S2-Notch cells express a higher level of Pkc98E compared to S2 cells (without Notch) and treatment with phorbol ester (TPA), a commonly used pharmacological activator of PKC, suppressed Pkc98E expression in S2-Notch cells (**Fig. 7A** **)**. Reduction in PKC level upon TPA treatment is a well-known negative regulatory response to PKC activation. Examination of Cactus level showed that P-Cactus increases in S2-Notch cells upon TPA treatment and this level is suppressed at high levels of TPA (**Fig. 7B** **;** note the concomitant increase in the un-phosphorylated form of Cactus at high TPA concentrations).

**Figure 8:**
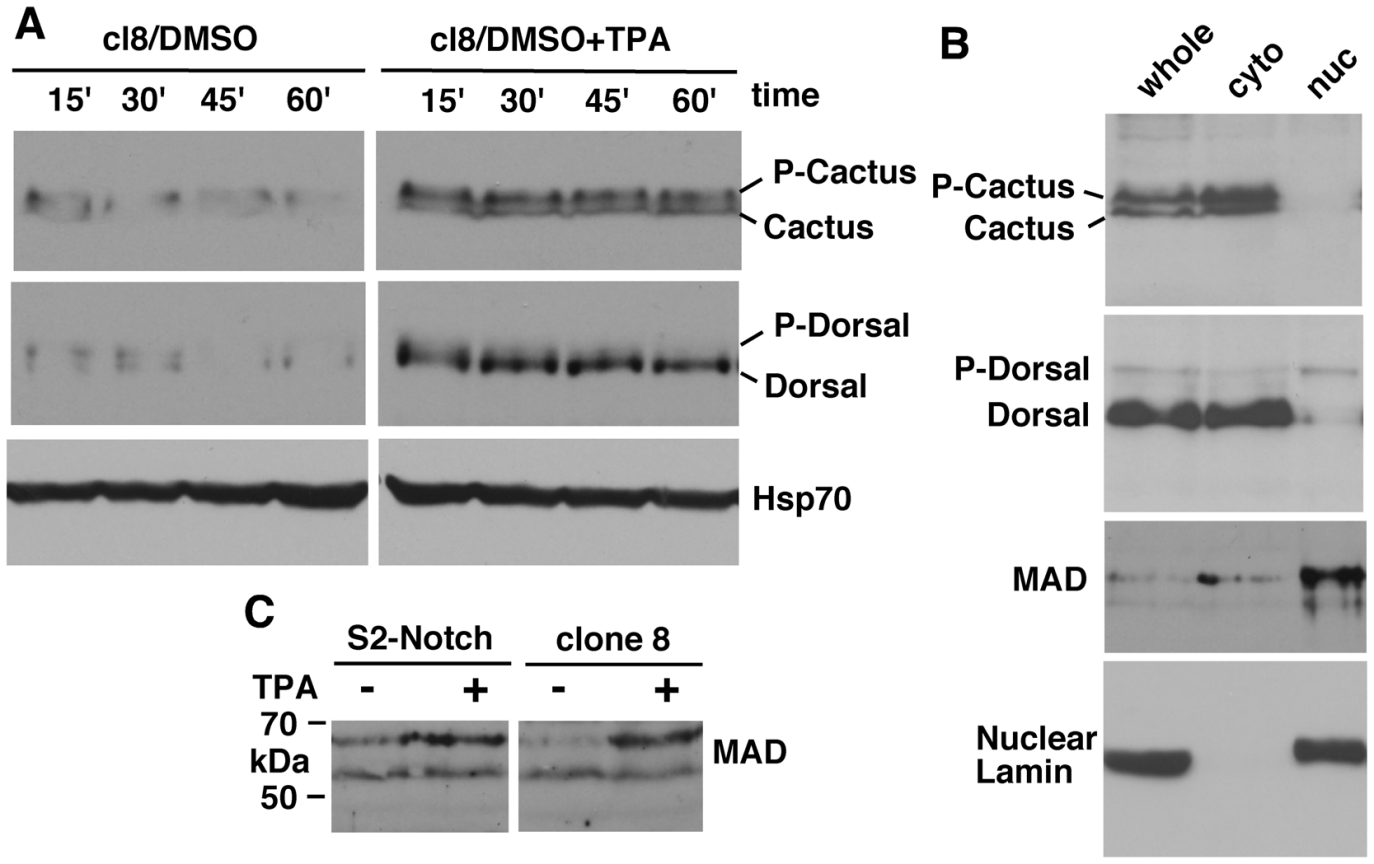
Cultured cells show the same responses observed in embryos with gain or loss of Notch activity. **A**. TPA treatment of cl8 cells (that express Notch endogenously) increases the level of P-Cactus and cytoplasmic Dorsal. The same samples were used for the three blots. **B**. Cell fractionation of cl8 cells treated with TPA shows the expected cytoplasmic localization for P-Cactus and nuclear localization for P-Dorsal and MAD proteins. The same samples were used for all four blots. Lamin is a nucleus specific marker. **C**. TPA activation of PKC in S2-Notch or cl8 cells increases the level of MAD protein. In all the experiments the same number of cells was used for each sample and the same amount of the sample was loaded in all lanes. TPA treatment was for 30 minutes. Experiment under **A** was repeated four times (with different time samplings), experiments under **B** was repeated three times, and experiments under **C** twice.

Our studies comparing the responses of S2-Notch cells (where transgenic Notch expression is induced through a heat shock promoter) and cl8 cells (where Notch is endogenously expressed) showed that the latter gave better results possibly because Notch is expressed close to physiological level. Therefore, we chose cl8 cells for more extensive analysis of Pkc98E activity. We found that Pkc98E could be activated in these cells for a sustained period by TPA treatment (at 500 nM concentration) and it resulted in increased expression of P-Cactus and cytoplasmic Dorsal (**Fig. 8A**). We performed cell fractionation studies and verified that P-Cactus is cytoplasmic and P-Dorsal is nuclear (**Fig. 8B**). We also found that both S2-Notch and cl8 cells treated with TPA increased the level of MAD and much of this MAD is in the nucleus as expected (**Fig. 8C** **and the third blot in** **Fig. 8B**). These experiments indicated that Pkc98E has the potential to be involved in the regulation of P-Cactus, Dorsal, and MAD in embryos, which we examined next.

### Pkc98E RNAi Embryos Manifest Phenotypes Linked to Loss of Toll and Dpp Signaling

**Figure 9:**
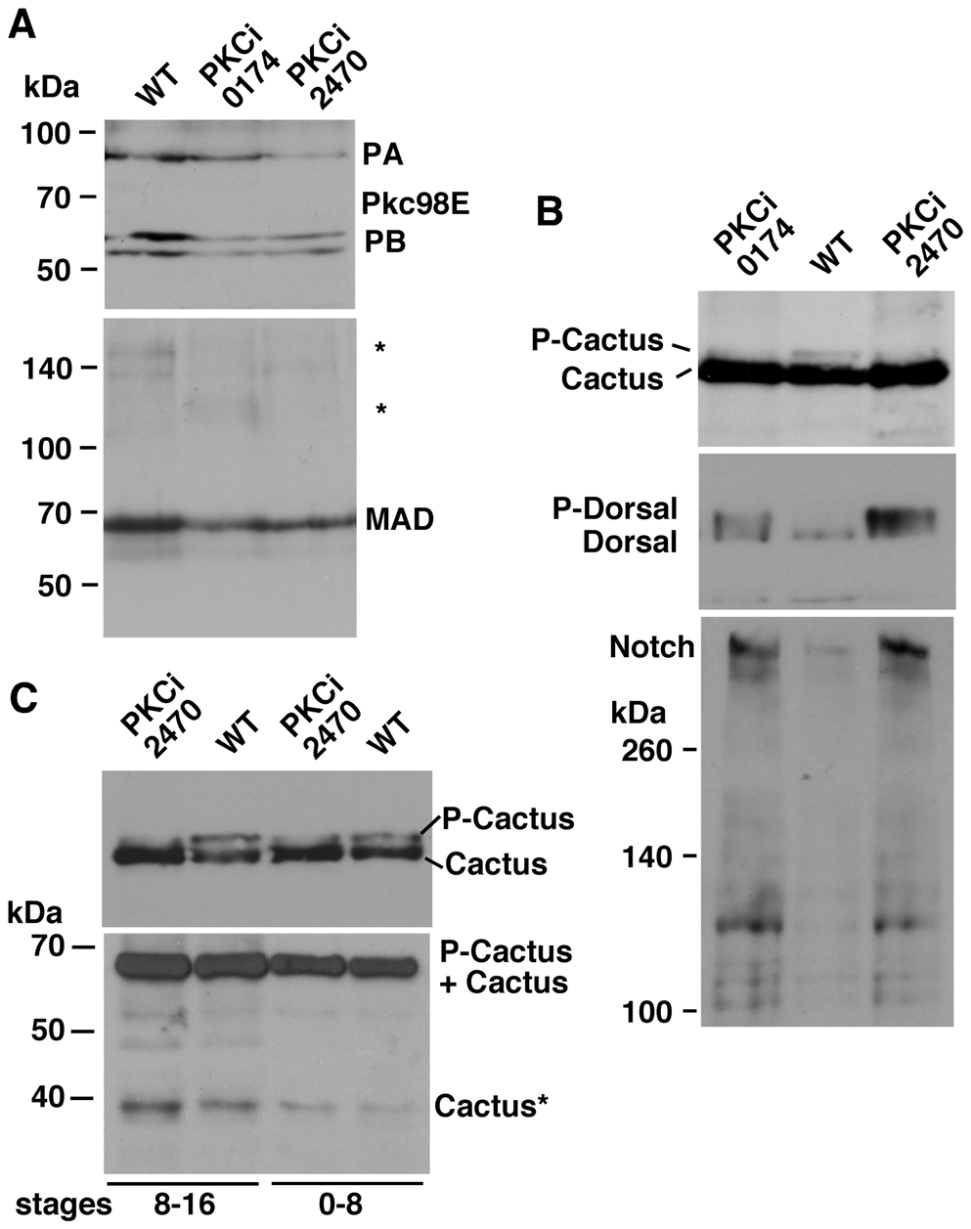
The effects of Pkc98E knock down in embryos. **A**. RNAi knock down of *Pkc98E* gene expression (short inverted repeat: PKCi 0174; long repeat: 2470) reduces the expression of Pkc98E proteins forms PA and PB and MAD. Extracts from stage 0–16 embryos are shown. The same sample was used for the two blots. * = background bands that additionally indicate protein loading is not an issue. **B**. Pkc98E knock down by RNAi reduces the level of P-Cactus and increases the level of Dorsal and Notch. Extracts from stage 0–16 embryos are shown. The same samples were used in the three blots. **C**. The level of the truncated Cactus form Cactus* is increased in PKCi embryos. Extracts from 8–16 stages (first two lanes) and 0–8 stages (last two lanes) are shown. The top blot based on 7.5% gel clearly shows P-Cactus and Cactus. These two bands merge in the 10% gel required to retain Cactus* (bottom blot). PKCi 0174 embryos show a similar response. In all the experiments, the same number of embryos was used for all samples and the same amount of the sample was loaded in each lane of a blot. Each result in these experiments was verified by at least three repetitions.

**Figure 10:**
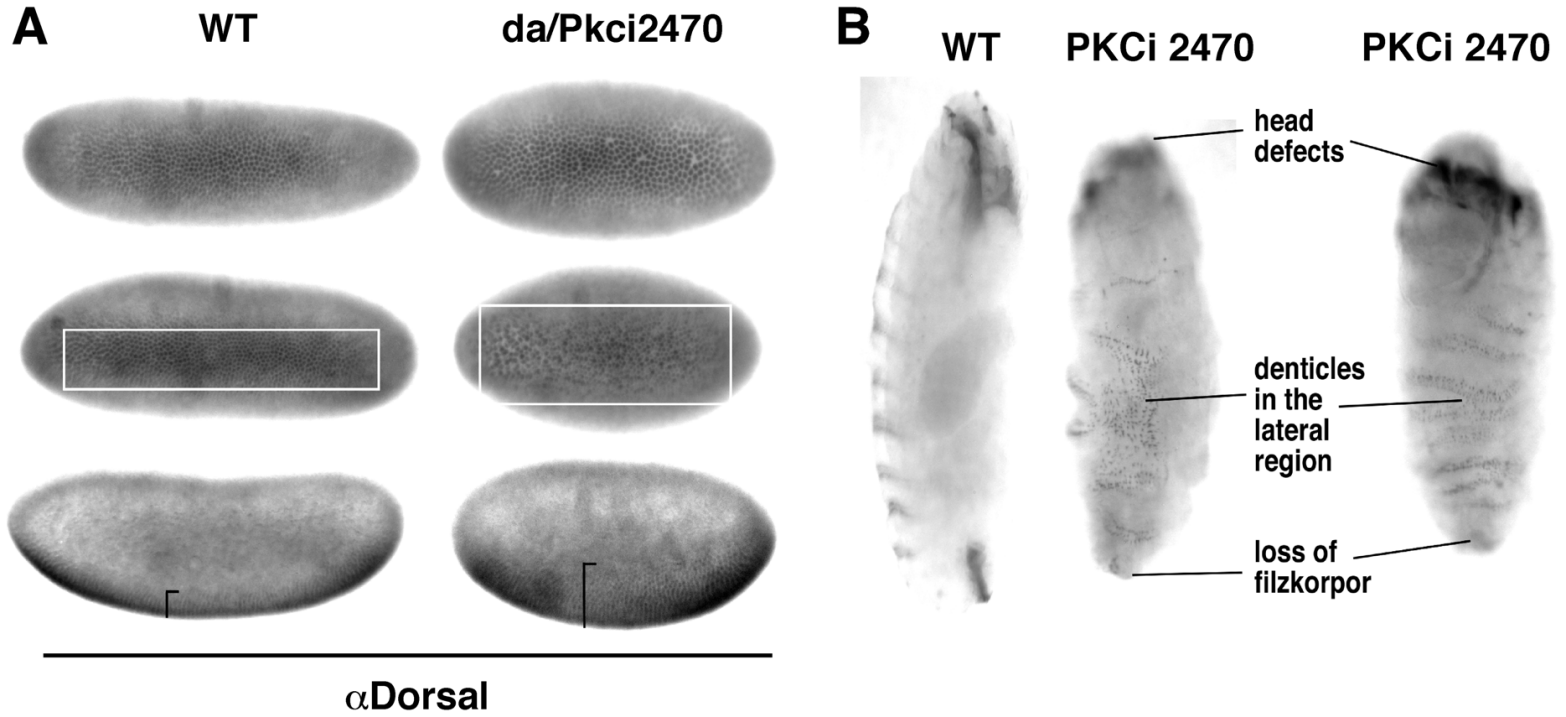
Pkc98E RNAi embryos manifest loss of Cactus and Dpp phenotypes. **A**. Nuclear Dorsal expression in the ventral region of Pkci embryos is irregular and expanded. Irregularity is apparent even at the very initial stages of dv axis formation (top row) and becomes pronounced at later stages (middle row). The last row shows embryos from a lateral perspective. **B**. Pkci embryos manifest denticles in the normally naked lateral regions and loss of head and filzkorpor structures that are associated with loss of Cactus and Dpp functions. The phenotypes were verified in two independently generated embryos for each of the two Pkci lines used.

To find out if loss of Pkc98E affects development along the dv axis in embryos, we relied on two UAS-RNAi lines (one uses a short hairpin, 0571, and the other, a long hairpin, 2470). We expressed these constructs using the Gal4 construct driven by the *daughterless* promoter (da-Gal4). This promoter is more or less ubiquitously active (including in the female germline). The constructs were functional; both 0571 and 2470 PKCi embryos expressed reduced levels of Pkc98E protein forms (**Fig. 9A**). Both PKCi embryos showed reduced levels of P-Cactus and MAD and increased level of P-Dorsal (**top two blots in** **Fig. 9B** **, top blot in** **Fig. 9C** **and the bottom blot in** **Fig. 9A**). We also detected increased level of the truncated Cactus* form that we had observed in Notch m^−^z^−^ embryos although not to the same degree (**the bottom blot in** **Fig. 9C**). One surprising result was the effect of loss of Pkc98E on Notch expression: Notch level increased dramatically (**the bottom blot in** **Fig. 9B**). We next examined the expression of nuclear Dorsal in PKCi embryos and found it to be very uneven and significantly expanded into the lateral regions (**Fig. 10A**). The ultimate proof that *Pkc98E* functions in the formation of the dv axis was provided by the embryonic phenotype shown in **Figure 10B**. It is a composite of the loss of Cactus and Dpp functions: denticles in the normally naked lateral regions, loss of head structures, and filzkorper (these are phenotypes associated with the loss of Toll and Dpp signaling; please confer allelic descriptions at FlyBase). The data described in this section support our cell culture data that Pkc98E up-regulates the levels of P-Cactus and cytoplasmic Dorsal and down-regulates the level of P-Dorsal. They further indicate that these Pkc98E regulations are inextricably linked to Notch expression and impact dv axis formation during Drosophila embryogenesis.

### Pkc98E RNAi Increases Notch Function in the Ventral Region of Drosophila Embryos

**Figure 11:**
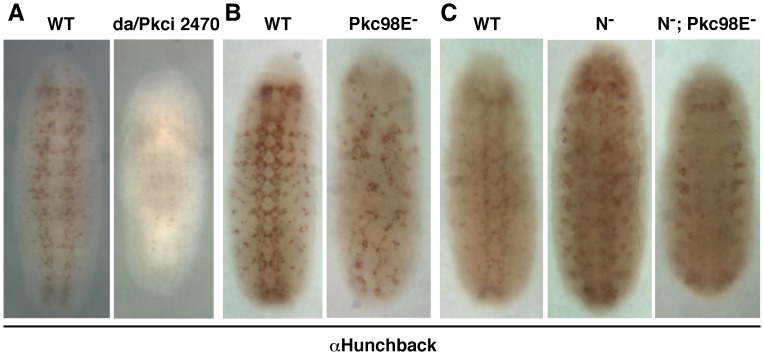
Loss of Pkc98E expression results in suppression of neurogenesis in the ventral region of embryos. **A**. RNAi knock down of Pkc98E expression (da/Pkci2470) significantly reduces not only the number of Hunchback expressing cells (i.e., neuronal cells) but also the amount of Hunchback in the few cells expressing it (i.e., reduced neuronal potential). The Pkci0174 line gave similar results. Embryos shown are at stage 9. **B**. Embryos homozygous for the null allele *Pkc98^Ef06221^* (Pkc98E^−^) also manifest reduced number of Hunchback expressing cells and reduced amount of Hunchback in cells. Embryos shown are at stage 10. **C**. The production of excess neuronal cells in the ventral region of zygotic Notch null embryos (N^−^) is blocked when embryos are simultaneously homozygous for the *Pkc98^Ef06221^* null allele (N^−^; Pkc98E^−^). Embryos shown are at stage 11. The phenotypes in **A** were verified in two independently generated embryos for each of the two Pkci lines used (the two alleles showed different penetrance and expressivity), the phenotypes in **B** were verified by most embryos at stage 10, and the phenotypes in **C** was verified in two independent embryo samplings from the same cross.

The data presented above show that loss of either Notch or Pkc98E results in reduced level of P-Cactus concomitant with increased level of P-Dorsal in embryos. The next question was whether Notch and Pkc98E function together or independently in embryos. Unfortunately this question cannot be addressed by standard genetic epistasis experiments because our data show that Notch promotes Pkc98E expression and Pkc98E suppresses Notch expression. However, the fact that Notch is over-expressed in PKCi embryos that manifest loss of Notch dv phenotypes (decreased P-Cactus/increased P-Dorsal) strongly argues that Notch and Pkc98E function together in dv axis formation. An important caveat to this argument is that Notch (over) expressed in PKCi embryos is the active form. If the over-expressed Notch is indeed the active form we expected to observe Notch gain on function phenotypes in processes that are not dependent on Pkc98E. All of our data indicate that canonical Notch signaling *per se* does not depend on PKC. Therefore, we studied neurogenesis in the ventral region that is based on canonical Notch signaling. We probed PKCi embryos with an antibody against Hunchback, which is a great marker for neurogenesis. We found that development of the CNS was strongly suppressed in PKCi embryos (**Fig. 11A**). Since suppression of neurogenesis at the proneural cluster stage could also lead to suppression of the CNS development, we probed the embryos for mRNA of the *achaete* gene, the marker for proneural clusters. Results showed that proneural clusters were robustly formed with *achaete* expression expanding into the lateral regions and persisting for longer time than in wild type embryos (**Fig. S4**). We next examined the CNS development in *Pkc98E* zygotic null embryos. *Pkc98E^f06221^* is an embryonic lethal, loss of function mutant allele of the *Pkc98E* gene generated by insertion mutagenesis. We found that neurogenesis was suppressed even in these *Pkc98E* zygotic null embryos (**Fig. 11B**). We then tested whether the neurogenic phenotype in *Notch* zygotic null embryos is suppressed by the *Pkc98E^f06221^* allele. We generated *Pkc98E* ^−^ *; N* ^−^ double mutant embryos and studied the CNS development using the Hunchback antibody. We found that the *N* ^−^ neurogenic phenotype was suppressed specifically in the ventral region of *Pkc98E* ^−^ *; N* ^−^ embryos (**Fig. 11C**). The neurogenic phenotype in the lateral regions persisted until the embryos ceased development suggesting that the suppression effect of the *Pkc98E^f06221^* allele was specific to the ventral region. These data indicated that loss of *Pkc98E* expression results in excess canonical Notch signaling in the ventral region, which is consistent with increased Notch expression in these embryos. Since *Pkc98E* ^−^ *; N* ^−^ embryos lack the zygotic product of the *Notch* gene it appears that the loss of *Pkc98E* expression resulted in increased function of the maternal Notch product and extended it to the lateral inhibition stage that is normally under the control of zygotic Notch product. We performed an additional test to confirm this finding. If loss of *Pkc98E* increased canonical Notch signaling, we expected wing notching in *Notch* heterozygous flies (produced because of reduced level of canonical Notch signaling) to be suppressed if these flies were also heterozygous for *Pkc98E*. Data shown in **Figure S5** show that wing notching is indeed suppressed by reduction in *Pkc98E* level. Thus, Notch is active in embryos lacking Pkc98E but just not in the lateral regions, where it apparently requires PKC98E activity to perform function related to P-Cactus and dv axis formation.

## Discussion

### The Notch Pathway Contributes to dv Axis Formation in Drosophila Embryos

Our data are obtained from embryos manifesting gain and loss of Notch activities. For gain, we used alleles of the *hephaestus* gene and the *Notch* gene that abrogate negative regulation of Notch expression [5], [6], [16], [35], [53], [67]–[69]. For loss, we used *Notch* and *Suppressor of Hairless* null alleles. In addition to embryos, we obtained data from Drosophila cultured cells. In one line Notch is expressed only from a heat shock inducible transgene, in the other it is expressed from the endogenous gene. All four data sets show remarkable concord: Notch activity promotes P-Cactus expression and suppresses nuclear P-Dorsal. This Notch activity impacts dv axis formation as with its loss the band of nuclear Dorsal expands into the lateral regions (in Notch m^−^z^−^ embryos) and with its gain (in heph m^−^z^−^ embryos) the lateral cells are apparently mis-specified as dorsal cells. We infer cell mis-specification rather than proliferation because at this stage of embryogenesis few cells divide.

The role of Cactus phosphorylation has been unclear in Drosophila [41], [43]. Our data show that Notch up-regulates P-Cactus, which is linked to suppressing the level of nuclear P-Dorsal. The underlying mechanism might involve Notch blocking endopeptidase cleavage of Cactus that generates the smaller Cactus* form or promoting the clearance of this form. Cactus* is observed at low levels in wild type embryos, indicating that it is produced even under normal circumstances as part of the turnover process or developmentally regulated degradation including degradation processes that are poorly understood [70]. However, it is also possible that Cactus* performs a hitherto unknown function. The possibilities discussed here are within the framework of the established model in the field, which is as follows. Association of Cactus and Dorsal in the cytoplasm stabilizes Cactus and retains Dorsal in the cytoplasm. Toll signaling degrades Cactus that enables phosphorylation of Dorsal and its nuclear translocation [41]–[48]. Our data fit this model. However, in our experiments changes in the levels of P-Cactus and P-Dorsal were linked except in very late stage embryos where we observe the P-Cactus response to Notch activation but we do not detect much Dorsal or P-Dorsal on western blots or in immuno-labeled embryos. However, this could be because P-Cactus affects other NFκB homologs (Dif or Relish) or has no functional consequences at these stages. Therefore, it remains possible that Notch activation results in blocking the phosphorylation of Dorsal that leads to accumulation of P-Cactus. In this scenario, P-Cactus could have a function reinforcing cytoplasmic retention of Dorsal (in association with the Dpp pathway) or an as of yet unknown function. Thus, it is possible that our data are revealing novel aspects that cannot be explained by the current model for dv axis formation in Drosophila embryos.

The effect of Notch activity on P-Cactus and Dorsal appears to be primarily restricted to the lateral regions. We have no evidence that it extends to the ventral region and have some evidence that it affects the dorsal region (e.g., the amnioserosa is small in Notch m^−^z^−^ embryos). Our data indicate that Notch activity (of any kind) is not required for P-Dorsal/nuclear Dorsal expression in Drosophila embryos as the level of the latter is higher and expanded into lateral regions of Notch m^−^z^−^ embryos. It appears that Notch activity can only suppress P-Dorsal/nuclear Dorsal expression, that too only in the lateral regions. It might oppose Toll signaling directly or indirectly through facilitation of Dpp signaling. Either way our data suggest that Notch activity in the lateral regions of the embryo might be important for generating the slopes of the opposing Toll and Dpp signaling gradients during formation of the dv axis. The role of canonical Notch signaling activity in the narrow band of mesectodermal cells is unclear. Based on our studies of Notch m^−^z^−^ embryos, heph m^−^z^−^ embryos, hsN^intra^/NICD embryos, and the expression of naturally produced dominant-negative forms of Notch [2], we think its role might be to regulate the invagination of the ventral mesodermal cells.

There is a puzzle embedded in our data. Restriction of Cactus-related Notch activity to the lateral regions in mid-to-late stage *hephaestus* and *N^nd1^* embryos suggests that the Drosophila embryo seems to maintain distinct ventral, lateral, and dorsal regions from the beginning to the end of embryogenesis even though different cell types occupy these regions at different embryonic stages. For example, once the mesodermal cells invaginate, the mesectodermal and neurorectodermal cells become the new ventral cells. This puzzle raises interesting questions. Are future changes to the position of cells incorporated into the initial process defining cell fates along the dv axis? Do factors in the perivitelline fluid provide continuous input to cells regarding their position along the dv axis? Do signaling based on cell-cell interactions re-calibrate positional information as embryogenesis proceeds along the dv axis?

### Canonical Notch Signaling is not Involved in Notch Function in the Lateral Regions

We were led to examine the role of Notch in dv axis formation by our observation of very high Cactus expression in the lateral regions of embryos that manifest gain in Notch activities (*heph^03429^*, *N^nd1^*). These embryos expressed very high levels of cell surface Notch and F-actin, and manifested severe phenotypes related to processes that depend on the proper development of the lateral epidermis and the dorsal amnioserosa (e.g., dorsal closure and cardiogenesis). Cactus was not over-expressed in the ventral region of the same embryos where neurogenesis was suppressed due to excess canonical Notch signaling. When canonical Notch signaling was specifically increased (through expression of N^intra^/NICD transgene or the classical mutant allele *l(1)N^B^*) the neurogenesis phenotype in the ventral region was reproduced but not any of the molecular or morphological phenotypes in the lateral or dorsal regions of *heph^03429^* and *N^nd1^* embryos. In fact, the levels of cell surface Notch and F-actin were suppressed and the opposite phenotypes were observed (e.g., suppression of amnioserosa, [35]). Therefore, we conclude that canonical Notch signaling is not involved in Cactus over-expression in the lateral regions of *heph^03429^* and *N^nd1^* embryos.

All of our experiments indicate that Notch accumulation at the cell surface in response to ligand binding is important for promoting the expression of P-Cactus. As *presenilin* and *kuzbanian* maternal and zygotic null embryos are known to produce phenotypes comparable to phenotypes in N(m^−^z^−^) embryos [71]–[73], it is quite likely they are also required for Notch activities both at the cell surface and in the nucleus, albeit in different ways. For example, Suppressor of Hairless is required for the stability of the Notch protein in the cytoplasm as well as for the transcriptional activity of N^intra^/NICD in the nucleus [12], [27], [74]. In a similar vein, it is possible that proper membrane anchoring and presentation of Notch at the cell surface requires association of Kuzbanian and Presenilin. Since there is no known way to keep all components required for N^intra^/NICD production and yet prevent its production [75], we obtained correlative data taking advantage of the difference in the time required for Notch stabilization at the cell surface in response to Delta binding and significant accumulation of N^intra^/NICD following proteolytic cleavages. In cultured cells, Notch accumulation at the cell surface happens immediately upon Delta binding while a significant amount of N^intra^/NICD is detected only after 30 minutes. In our experiments, we observe a significant increase in P-Cactus level or co-expression of Cactus with Notch at the cell surface only within the first 20 to 30 minutes of Delta treatment. Thus, the increase in P-Cactus expression appears to be closely linked to Notch accumulation at the cell surface rather than to N^intra^/NICD accumulation.

In our view the *Pkc98E* loss of function embryos provide the most compelling data against canonical Notch signaling being involved in Notch regulation of P-Cactus. The anti-neurogenic phenotype (suppression of CNS development) that manifests in the ventral region of these embryos indicates gain in canonical Notch signaling but these embryos also manifest loss of Notch function phenotypes related to P-Cactus and P-Dorsal expression in the lateral regions. Samples of *Pkc98E* ^−^ embryos taken at different embryogenesis stages show that the loss of Notch function phenotype related to P-Cactus and P-Dorsal expression includes the stages when the gain of canonical Notch signaling phenotype (suppression of neurogenesis) manifests in the ventral region. Thus, the loss and gain of Notch function dichotomy between the two regions cannot be due to difference in timing of the developmental processes involved. Furthermore, the expansion of nuclear Dorsal into the lateral regions of N(m^−^z^−^) embryos is not due to loss of canonical Notch signaling (in the mesectoderm) because an excess amount of this signaling (in PKCi embryos) does not block the expansion. Finally, if Notch activity were involved only in mesectoderm formation along the dv axis, it would not be consistent with the expansion of the dorsal fate cells in embryos manifesting gain of Notch activity; expansion of ventral or lateral cells would have been consistent. Thus, a novel non-canonical Notch signaling at the cell surface is involved in the regulation of P-Cactus expression and cell fate specification in the lateral regions of the embryo.

### Pkc98E is Involved in Notch Activity in the Lateral Regions of Drosophila Embryos

Loss of *Notch* gene expression or reduction in *Pkc98E* gene expression yields the same molecular and morphological dv axis phenotypes in embryos, which implies that both *Notch* and *Pkc98E* function in the dv axis formation. The obvious question is whether they function together. Five sets of data indicate that they do: (1) positive regulation of Pkc98E level by Notch in embryos; (2) negative regulation of Notch level by Pkc98E in embryos; (3) Notch promotion of Pkc98E expression and activity in cultured cells; (4) pharmacological activation of PKC in cultured cells that reproduces the effect of Notch and Delta on P-Cactus, P-Dorsal, and MAD; and (5) Notch loss of function dv axis related molecular phenotypes in *Pkc98E* RNAi embryos even though Notch is over-expressed and is functional. In this regard, the effect of the reduction in *Pkc98E* expression on Notch protein expression is significant because in our experience only genes that are directly involved in Notch functions have such an effect (e.g., *Delta* or *Suppressor of Hairless*). Thus, our data indicate that Notch and Pkc98E function together and rule out other explanations such as *Notch* being downstream of *Pkc98E*, *Pkc98E* being downstream of *Notch*, and *Notch* and *Pkc98E* functioning independently.

It is also significant that the reduction in *Pkc98E* expression results in increased Notch function in the ventral region, as evidenced by suppression of neurogenesis. This effect, presumably due to extension of the function of maternally contributed Notch, indicates that even though *Pkc98E* is not required for canonical Notch signaling *per se* its normal expression in the ventral cells limits Notch availability for this signaling. Understanding how Pkc98E limits Notch availability to canonical Notch signaling in the ventral region of embryos and how N^intra^/NICD is blocked from translocating to the nucleus in the lateral regions might hold the key to understanding how the Notch-Pkc98E activity is suppressed in the ventral region but promoted in the lateral regions. Since PKC is activated only when it is recruited to the cell surface, it is quite possible that Notch activation by Delta recruits Pkc98E to the cell surface. It is possible that this recruitment is limited in cells of the ventral region but facilitated in cells of the lateral regions (except those differentiating the peripheral nervous system) to activate the non-canonical Notch signaling mechanism at the cell surface. In mammals, NICD that is the equivalent of N^intra^ and the mediator of canonical Notch signaling is known to bind the MAD homolog, SMAD, and function with it synergistically in the nucleus [for example, 76–78]. Our studies raise the additional possibility that the Notch intracellular domain might synergistically function with MAD at the cell surface or in the cytoplasm, in opposing generation of nuclear P-Dorsal through up-regulation of P-Cactus level.

**Figure 12:**
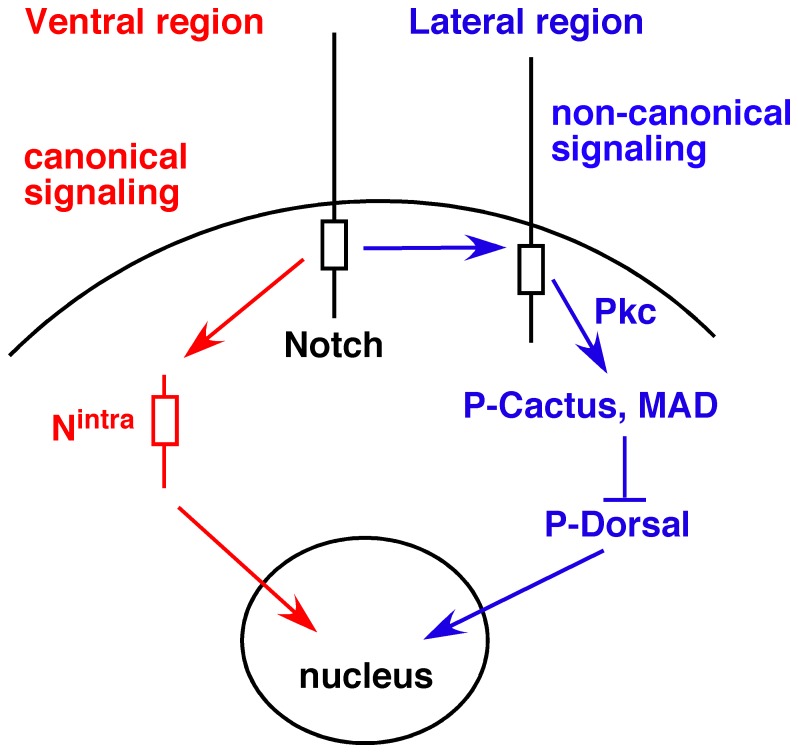
A working model for Notch functions along the dv axis of Drosophila embryos. Cells in the ventral region (including the mesectodermal cells at earlier stages) have the potential for canonical Notch signaling, which is blocked (e.g., in mesodermal cells) or realized (e.g., in neuroectodermal cells) in a stage-dependent manner. Most cells in the lateral regions (possibly excepting those that differentiate the peripheral nervous system) generate non-canonical Notch signaling at the cell surface. Factors that promote or suppress N^intra^ production or its nuclear transport might determine whether canonical or non-canonical Notch signaling predominates in a region.

Based on our data and interpretations presented above we propose the model presented in **Figure 12** for Notch functions along the dv axis of Drosophila embryos. The ventral region (including the mesectoderm) has the potential for canonical Notch signaling that is realized during gastrulation (mesoderm invagination) or neurogenesis. The lateral regions activate the non-canonical Notch activity linked to Pkc98E, which up-regulates P-Cactus and MAD expression. Since too much of it results in excess dorsal fate and too little in excess ventral fate, it appears that Notch-Pkc98E activity is used to form the slopes of the opposing Toll and Dpp signaling gradients along the dv axis. The intriguing question for future studies is how interactions between neighboring cells, which are known to yield variable and statistical outcomes for the cells involved (for example, lateral inhibition during the CNS development), can be used to generate the monotonic slope of a gradient of regulatory factors.

### Significance of Notch-Pkc98E Connection to Drosophila Embryogenesis

For more than 15 years we have been trying to understand non-canonical Notch signaling activities in embryogenesis, specifically in the development of the epidermis and the CNS in the embryo [2], [8], [12], [16], [34], [35]. Many others have also described various Notch activities that do not involve canonical Notch signaling [28]–[33]. It was not clear whether all these activities are based on different non-canonical mechanisms or a single mechanism. Based on the knowledge gained from this study we think that most, if not all, non-canonical Notch signaling instances in the fly might be based on Notch-Pkc98E activity at the cell surface. In this regard Notch might be similar to Armadillo/β-catenin, which performs distinct functions at the cell surface (cell adhesion) and in the nucleus (transcriptional activity). The data presented here and in our last paper [35] contain pivotal clues for dissecting the underlying mechanism of Notch signaling activity at the cell surface. There are indications that this mechanism might also involve the truncated Notch molecules that are naturally produced during embryogenesis and behave as dominant-negative molecules for canonical Notch signaling [2], [8], [12], [19]. For example, the Notch intracellular domain might be cleaved at intracellular sites (to produce NΔCterm and NΔI) to prevent the production of the full length N^intra^/NICD and its migration away from the cell surface (and into the nucleus). NΔCterm and NΔI accumulate specifically in the pole (germ) cells that form at the posterior end of the embryo [2]. This accumulation might be significant in the light of Hephaestus accumulation in these cells and its repression of translation of *oskar* mRNA [79]. This *hephaestus* function might also involve Notch regulation, as Notch and Delta activities across the germ and somatic (follicle) cells are critical for establishing anterior-posterior axis during oogenesis [80]–[83]. We have not explored the elaborate protein modification and trafficking processes the Notch protein is subjected to during embryogenesis [3], [4], [20], [22]. It would not be surprising if some of these processes are related to regulation between nuclear and cell surface Notch signaling activities.

Beside the potential for assimilating disparate pieces of information in the field into a coherent mechanism, the connection between Notch-Pkc98E activity and dv axis formation has the potential to identify the function of the non-canonical Notch activity. In our view the crucial clue is the effect of Notch-Pkc98E activity on Dpp signaling. Processes at the interface between cell surfaces and the extracellular matrix critically regulate the formation of Dpp signaling gradient along the dv axis. For example, factors in the extracellular space are responsible for activating or modifying Dpp morphogen [36]–[43]. It is possible that the function of all non-canonical Notch activities reported to be involved in cell adhesion, cell migration, and epithelia remodeling [35], [28]–[33] is to regulate developmental potential in the intercellular space. This function would be complementary to the function of canonical Notch signaling that regulates developmental potential from within the cell (through activity in the nucleus).

### Significance of Notch-PKC Connection to Mammalian Processes

Notch functions are highly conserved between Drosophila and mammals. In fact, the basic framework for the study of canonical Notch signaling in mammals was developed based on data from Drosophila embryos. Many non-canonical Notch functions have been described from various mammalian systems and the majority relates to actin or extracellular matrix processes. The mechanism(s) underlying these non-canonical functions are unknown. Our data could serve as guides for deciphering these mammalian non-canonical Notch mechanism(s). If canonical and non-canonical Notch mechanisms are connected in mammals the way they are in Drosophila embryos (e.g., neurogenesis) it would have additional significance since both mechanisms are implicated in a wide array of human diseases including developmental defects, cardiovascular diseases including strokes, Alzheimer’s and dementia, Down syndrome, immune deficiency, prion diseases, and a variety of cancers [84]–[93]. All these diseases also involve PKC, Toll/NFκB, and Dpp/BMP signaling [36]–[40], [61]–[65], [94]–[105].

## Materials and Methods

### Flies

*N^nd1^*, N^nd3^, *heph^03429^*, *yellow white* (*yw*), *N^264–47^ FRT101*, *Su(H)^Δ47^ FRT40A*, *daughterless Gal4* (*daGal4*) are the same stocks used in [2], [5], [6], [12], [16], [21], [35], [53]. *w; FRT82B heph^03429^/TM6B placz (ry^+^)* was obtained from Anne Ephrussi [79]. UAS-cactus RNAi lines HMS00084 and HM04020 (stock numbers 34775, 31713), Pkc98E RNAi lines GL00174 and JF02470 (stock #s 35275, 29311), Pkc null line Pkc98E^f06221^/TM6 Tb^1^ (stock # 18950), and lines for generating maternal-zygotic null embryos: ovo^D1^ FRT101/C(1)Dx, hsFlp (stock # 1813), ovo^D1^ FRT40A/Cyo (stock# 2121), FRT82B/TM3 (stock # 2149), hsFlp; Dr1/TM3 Sb^1^ (stock # 26902), hsFlp; Adv^1^/Cyo (stock # 6) were obtained from the Bloomington Stock Center, Indiana University, Bloomington, Indiana, USA. Standard fly procedures were followed for staging, identification of genotypes (using the green or blue balancer chromosomes), and generation of maternal and zygotic null embryos [49], [106], [107]. Specific details are described in our previous papers [2], [12], [35].

### Cultured Cells

All the Drosophila cultured cell lines used in this study and details of the procedures used for studying Notch activities have been previously described [8], [9],[12],[16],[19],[26],[35],[54]. Briefly, exponential phase cells growing in fresh medium for two days were used for all cell types and in all experiments the incubation times (after Delta or phorbol ester treatment) were between 15 and 20 minutes. PKC activity was induced using Phorbol 12-Myristate 13-Acetate (12-O-tetradecanoylphorbol 13-acetate, TPA) from Sigma (Product # P1585) following their suggested protocol.

### Molecular Biology

Standard western blotting, northern blotting, immuno-labeling, and RNA *in situ* procedures were followed [108]–[110]. Specific details of the procedures used are described in our previous publications [2], [5], [6], [8], [12], [16], [19], [26], [35], [54]. The only note we would like to add and emphasize is that the best protein extraction procedure for these experiments is the one that yields molecules closest to their physiological states at the time of extraction: crushing in 1X Laemmli buoffer. Cactus, Dorsal, and Drosophila Lamin Dm0 mouse monoclonal antibodies were obtained from Developmental Studies Hybridoma Bank (DSHB) at the University of Iowa, Iowa City, Iowa, USA (3H12, 7A4, and ADL67.10, respectively); Notch polyclonal antibodies made in hamster or Rabbit used are previously described [2], [9]; Pkcδ antibodies made in rabbit that recognize Drosophila Pkc98E protein were obtained from Santa Cruz Biotechnology, Inc. (sc-213); Hunchback antibody was a gift from Paul MacDonald; Dpp (rabbit) and P-MAD (rabbit) antibodies were gifts from Armen Manoukian and Allen Laughon, respectively. Secondary antibodies were obtained from Jackson Laboratories or Molecular Probes/Invitrogen. For assessment of Cactus, Dorsal, Pkc98E, or MAD protein in western blots, 7.5% or 10% SDS-PAGE was used; for Notch 6% SDS-PAGE was used. Immuno-labeling and RNA *in situ* experiments were repeated at least two times and embryos were sorted into developmental stage series. Phenotypes were verified in at least five embryos of identical stage.

### Bioinformatics

Data, analytical tools, and external links accessible at the FlyBase, University of California at Santa Cruz (UCSC) Genome Browser, and the National Center for Biological Information (NCBI) websites were used. Outputs were used without modification, except cropping outside of the region of interest.

### Microscopy and Imaging

Embryos labeled using Horse Radish Peroxidase or Alkaline Phosphate based-detection system were imaged using an upright SMZ1500 stereoscope with a Spot RT Slider CCD camera. The same setting was used to image embryos that were directly compared. For confocal microscopy, Zeiss 510 Confocal Laser Scanning Microscope was used. Images obtained were processed using the Adobe Photoshop program and assembled into panels using the Canvas program (Deneba/ACD). Any adjustment to contrast or brightness was applied to the whole image and applied at identical values to all images that were directly compared.

## Supporting Information

Figure S1 **Notch level in wild type (yw) and gain of function N^nd1^ and N^nd3^ embryos.** Embryos were collected over 3 hours and incubated for 1-hour at 30°C. The same number of embryos was used to make protein extracts and the same amount of the extract was loaded in each lane. The level of hsp70 protein confirms that this method results in equal loading of total proteins.
(TIF)

Figure S2 **Cell surface Notch accumulation and N^intra^/NICD accumulation affect the expression of different genes.** **A**. Expression of *ovo-shavenbaby* (svb) mRNA is very high immediately upon mixing S2-Notch and S2-Delta cells (lanes 1 and 3 compared with lanes 2 and 4, respectively). Its level reaches the background level for S2-Notch cells after 45 minutes (lanes 5–6). On the other hand, expression of *E(spl)C m3* mRNA is low immediately upon mixing and becomes high 45 minutes later (lanes 1–6, middle blot). Data shown in **A** are from northern blots. The same blot was probed with different genes. rp49 = mRNA loading control. N = S2-Notch; Dl = S2-Delta cells; DlΔI = Delta with the transmembrane domain but without the carboxyl terminal intracellular domain; S2 = S2 cells expressing neither Notch nor Delta. S2-DlΔI cells that activate Notch as well as S2-Delta cells is used to show that signaling is generated through the Notch intracellular domain (i.e., in S2-Notch cells). Min = minutes of incubation (centrifugation to pellet cells and lysis took between 3–5 minutes). Note that the differential response of svb and E(spl)C on the same blot obviates the need for rp49 control, which is included just to show that similar amounts of RNA are present in lanes 7–10. **B**. N^intra^/NICD protein level is at the background level immediately after mixing S2-Notch and S2-Delta cells (0 min) and significantly high after 45 min. Data shown are from western blots using the same cell populations used for northern blots in **A**. Hsp70 = total protein loading control. We have previously shown that Notch is stabilized at the cell surface (in clusters) immediately upon treatment with S2-Delta cells [16], [19], [35].
(TIF)

Figure S3 **Shared conserved domains in Cactus (first sequence from top), dCreb 17b (second sequence), actin 5C (third sequence), and** ***Pkc98E*** **(fourth sequence) that map to the Ankyrin repeats of Notch (fifth sequence).** Alignment was generated using the COBALT program (NCBI). Red line marks region of conservation. There was no shared conserved domain of any length on either the amino terminal or carboxyl terminal of the region marked by the red line.
(TIF)

Figure S4 **Expression of** ***achaete*** **mRNA, which defines proneural clusters, in wild type (yw) and Pkc98E RNAi (da/Pkci2470) embryos.** Proneural clusters were robustly formed, with *achaete* expression expanding into the lateral regions of Pkci embryos (compare the first embryo in **B** with the first embryo in **A**) and persisted for a longer time than in wild type embryos (compare stage the last embryos in **A** and B). Note that while *achaete* mRNA expression has almost disappeared by stage 10 in the wild type embryos, it is still expressed at a high level in stage 11 Pkci embryos. The same data were obtained with da/Pkci0174 embryos.
(TIF)

Figure S5 **Heterozygosity for** ***Pkc98E*** **suppresses the wing notching phenotype of** ***Notch*** **heterozygous flies.** **A**. Representative wings of flies that are double heterozygotes for *Notch* and *Pkc98E* genes and flies that are double heterozygotes for *Notch* and an unrelated gene *Tubby* (Tb) on the TM6 balancer chromosome. These flies were siblings from the same cross. **B**. Table showing the distribution of severity of wing notching in *Notch*; *Pkc98E* and *Notch*; *Tubby* double heterozygous flies derived from the same cross.
(TIF)
