# Supplementary figures and images for: Notch and PKC Are Involved in Formation of the Lateral Region of the Dorso-Ventral Axis in Drosophila Embryos
Source: PLoS One. 2013 Jul 4;8(7):e67789. doi: 10.1371/journal.pone.0067789 (PMC3701627; doi:10.1371/journal.pone.0067789)

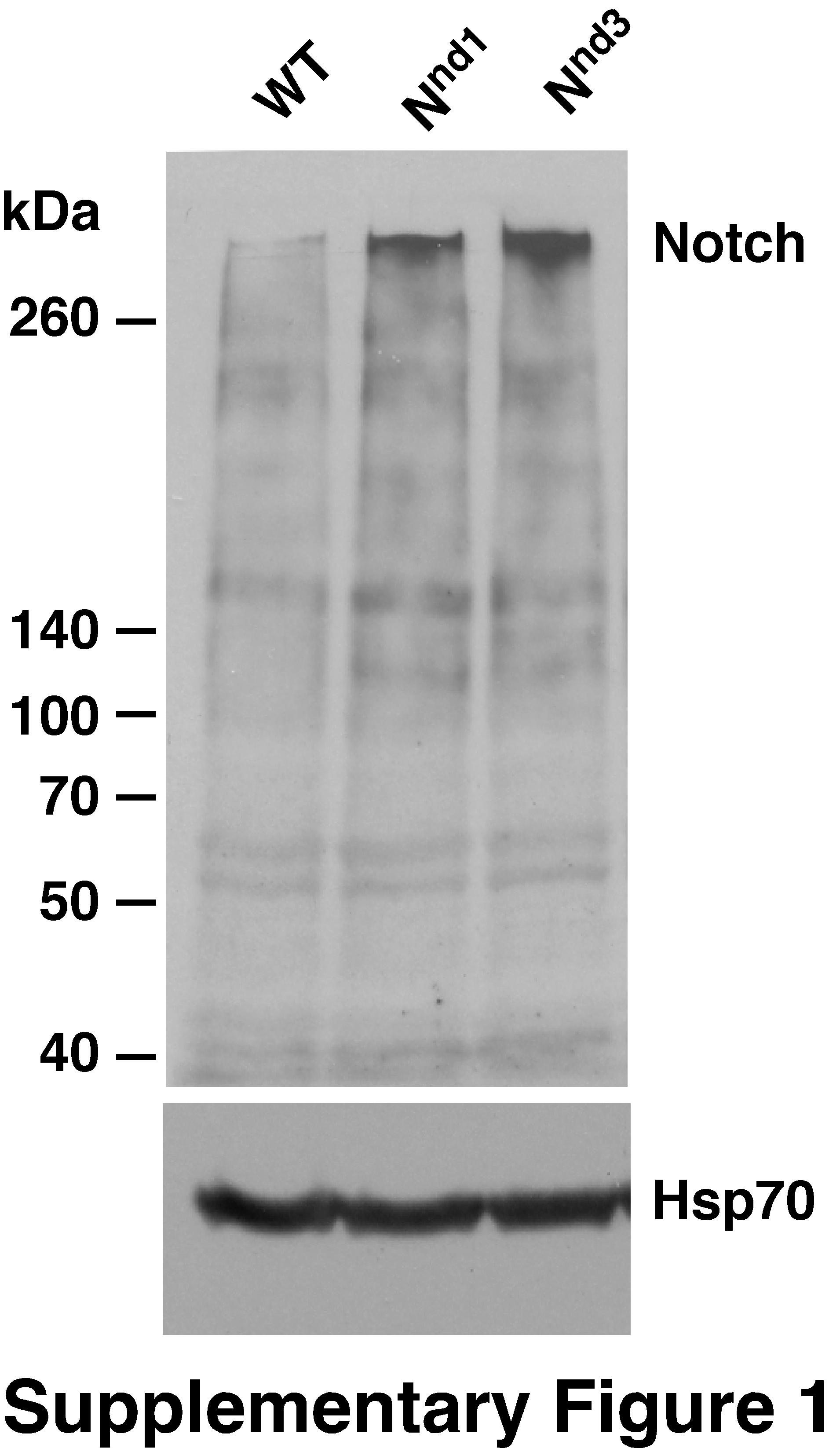

Supplement: Figure S1 — Notch level in wild type (yw) and gain of function Nnd1 and Nnd3 embryos. Embryos were collected over 3 hours and incubated for 1-hour at 30°C. The same number of embryos was used to make protein extracts and the same amount of the extract was loaded in each lane. The level of hsp70 protein confirms that this method results in equal loading of total proteins. (TIF) [file pone.0067789.s001.tif]

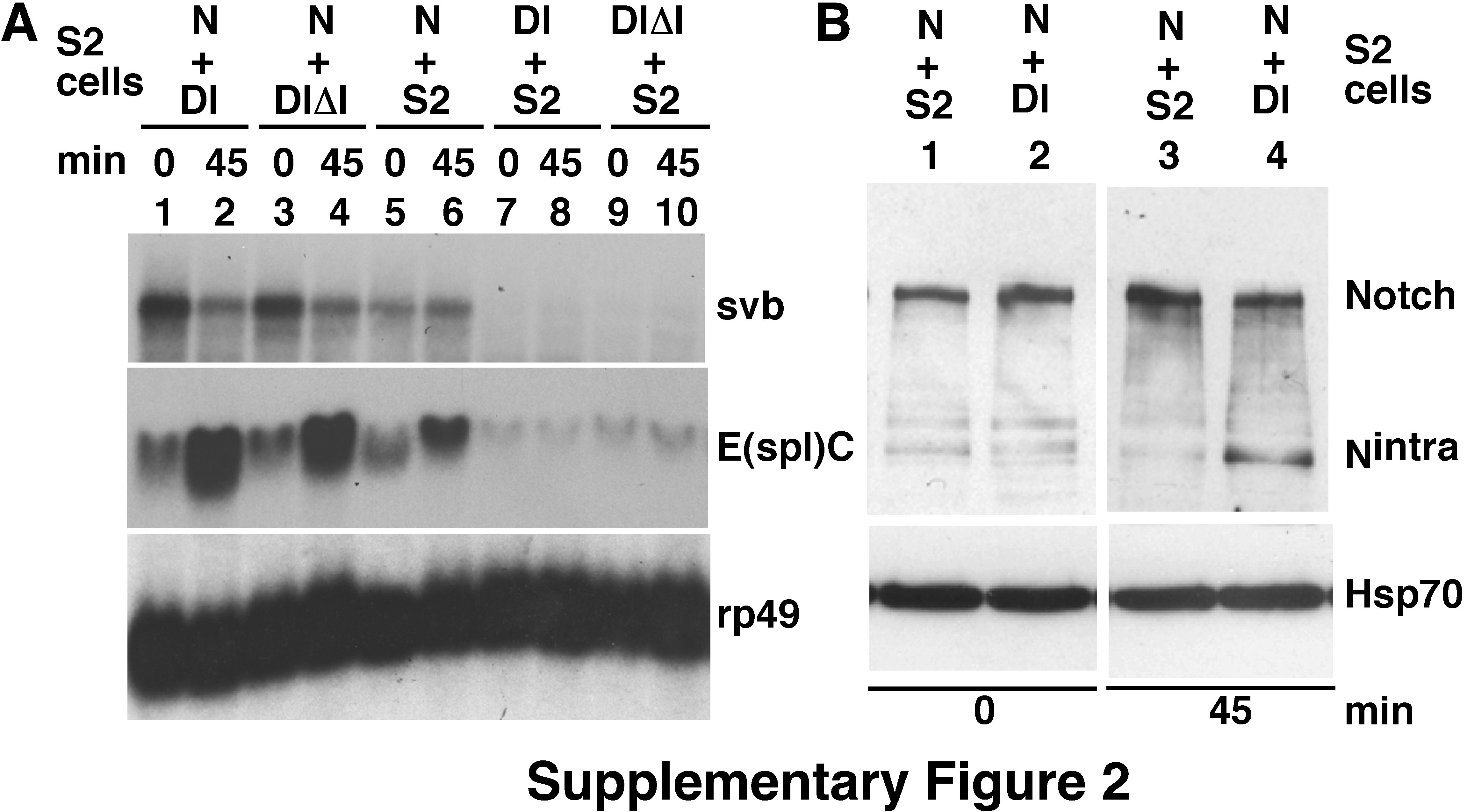

Supplement: Figure S2 — Cell surface Notch accumulation and Nintra/NICD accumulation affect the expression of different genes. A. Expression of ovo-shavenbaby (svb) mRNA is very high immediately upon mixing S2-Notch and S2-Delta cells (lanes 1 and 3 compared with lanes 2 and 4, respectively). Its level reaches the background level for S2-Notch cells after 45 minutes (lanes 5–6). On the other hand, expression of E(spl)C m3 mRNA is low immediately upon mixing and becomes high 45 minutes later (lanes 1–6, middle blot). Data shown in A are from northern blots. The same blot was probed with different genes. rp49 = mRNA loading control. N = S2-Notch; Dl = S2-Delta cells; DlΔI = Delta with the transmembrane domain but without the carboxyl terminal intracellular domain; S2 = S2 cells expressing neither Notch nor Delta. S2-DlΔI cells that activate Notch as well as S2-Delta cells is used to show that signaling is generated through the Notch intracellular domain (i.e., in S2-Notch cells). Min = minutes of incubation (centrifugation to pellet cells and lysis took between 3–5 minutes). Note that the differential response of svb and E(spl)C on the same blot obviates the need for rp49 control, which is included just to show that similar amounts of RNA are present in lanes 7–10. B. Nintra/NICD protein level is at the background level immediately after mixing S2-Notch and S2-Delta cells (0 min) and significantly high after 45 min. Data shown are from western blots using the same cell populations used for northern blots in A. Hsp70 = total protein loading control. We have previously shown that Notch is stabilized at the cell surface (in clusters) immediately upon treatment with S2-Delta cells [16], [19], [35]. (TIF) [file pone.0067789.s002.tif]

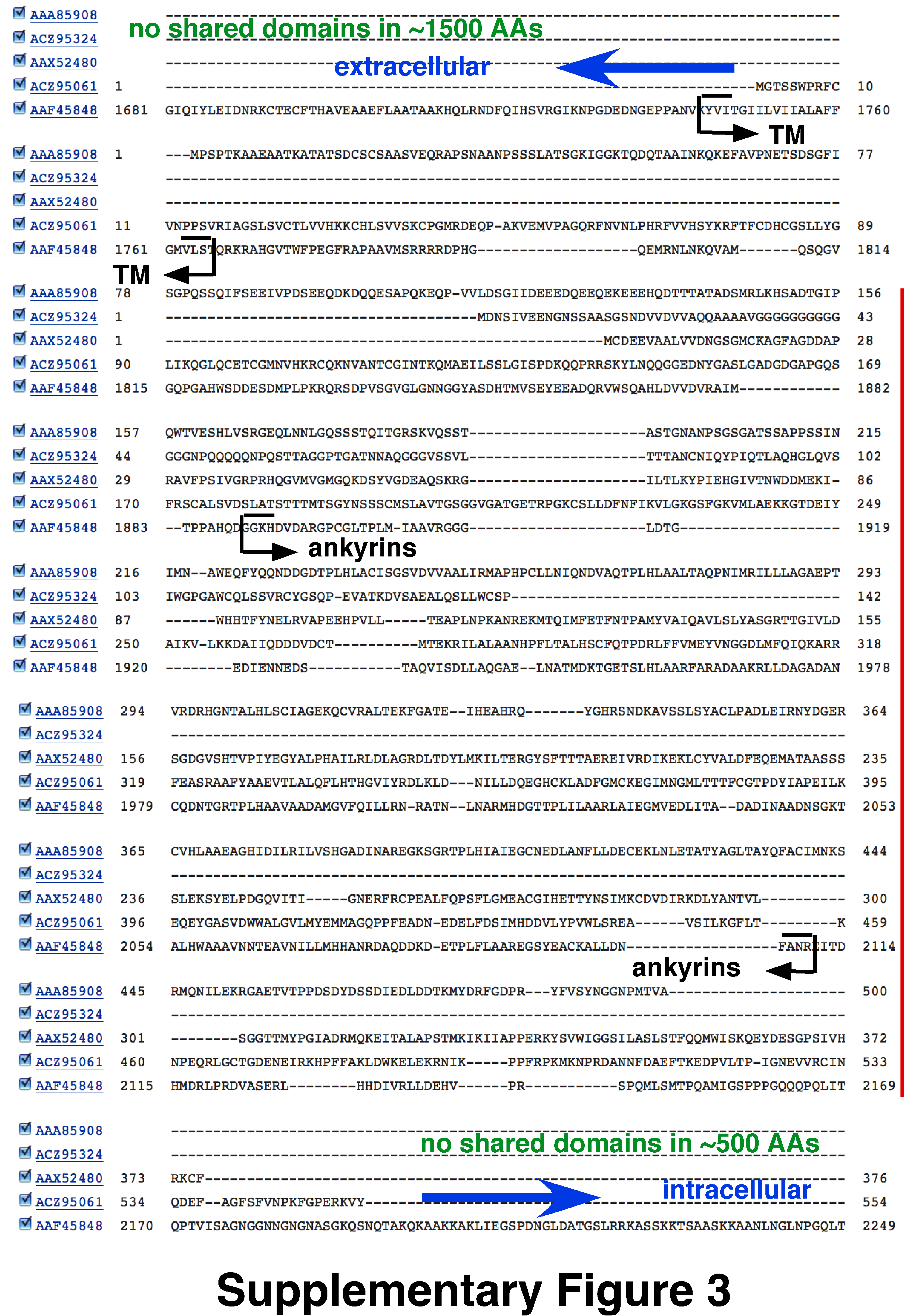

Supplement: Figure S3 — Shared conserved domains in Cactus (first sequence from top), dCreb 17b (second sequence), actin 5C (third sequence), and Pkc98E (fourth sequence) that map to the Ankyrin repeats of Notch (fifth sequence). Alignment was generated using the COBALT program (NCBI). Red line marks region of conservation. There was no shared conserved domain of any length on either the amino terminal or carboxyl terminal of the region marked by the red line. (TIF) [file pone.0067789.s003.tif]

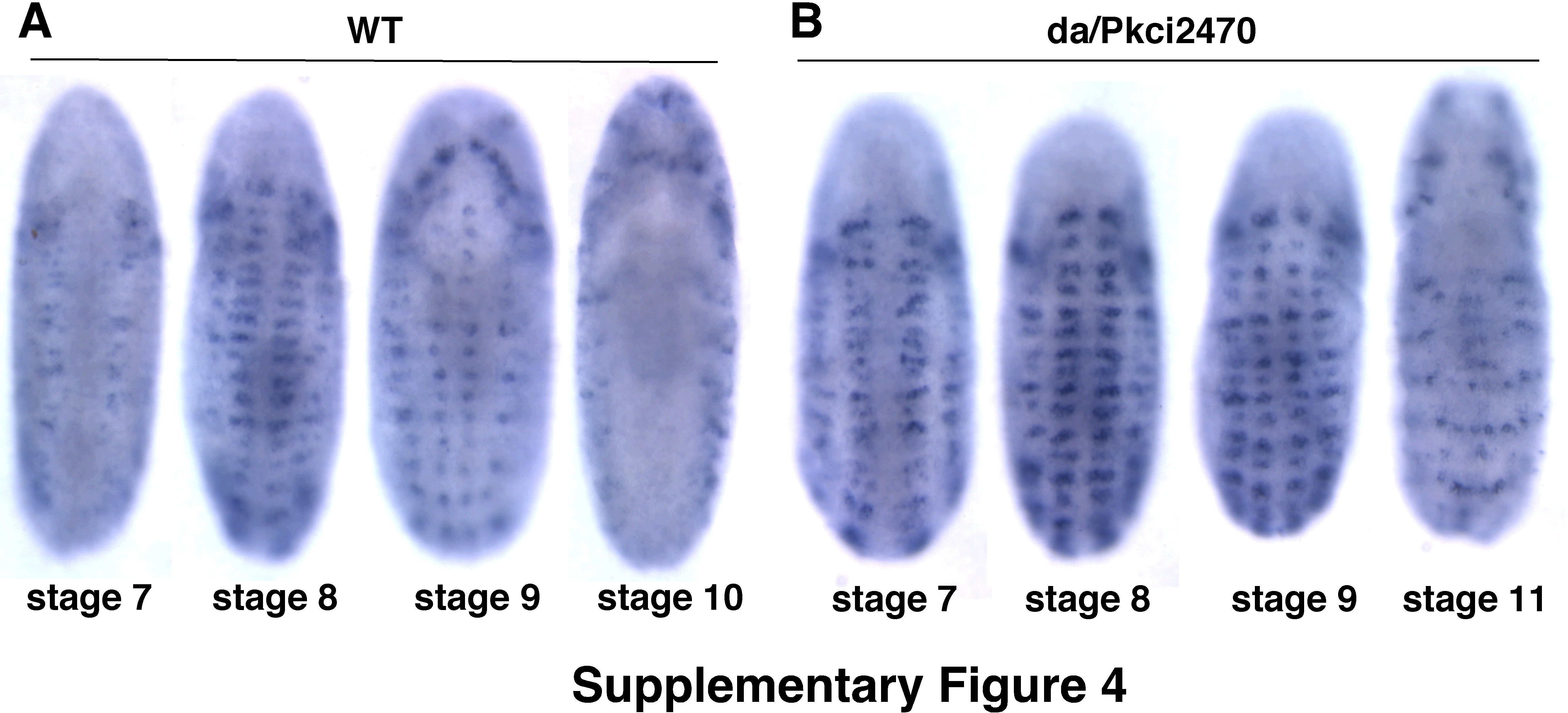

Supplement: Figure S4 — Expression of achaete mRNA, which defines proneural clusters, in wild type (yw) and Pkc98E RNAi (da/Pkci2470) embryos. Proneural clusters were robustly formed, with achaete expression expanding into the lateral regions of Pkci embryos (compare the first embryo in B with the first embryo in A) and persisted for a longer time than in wild type embryos (compare stage the last embryos in A and B). Note that while achaete mRNA expression has almost disappeared by stage 10 in the wild type embryos, it is still expressed at a high level in stage 11 Pkci embryos. The same data were obtained with da/Pkci0174 embryos. (TIF) [file pone.0067789.s004.tif]

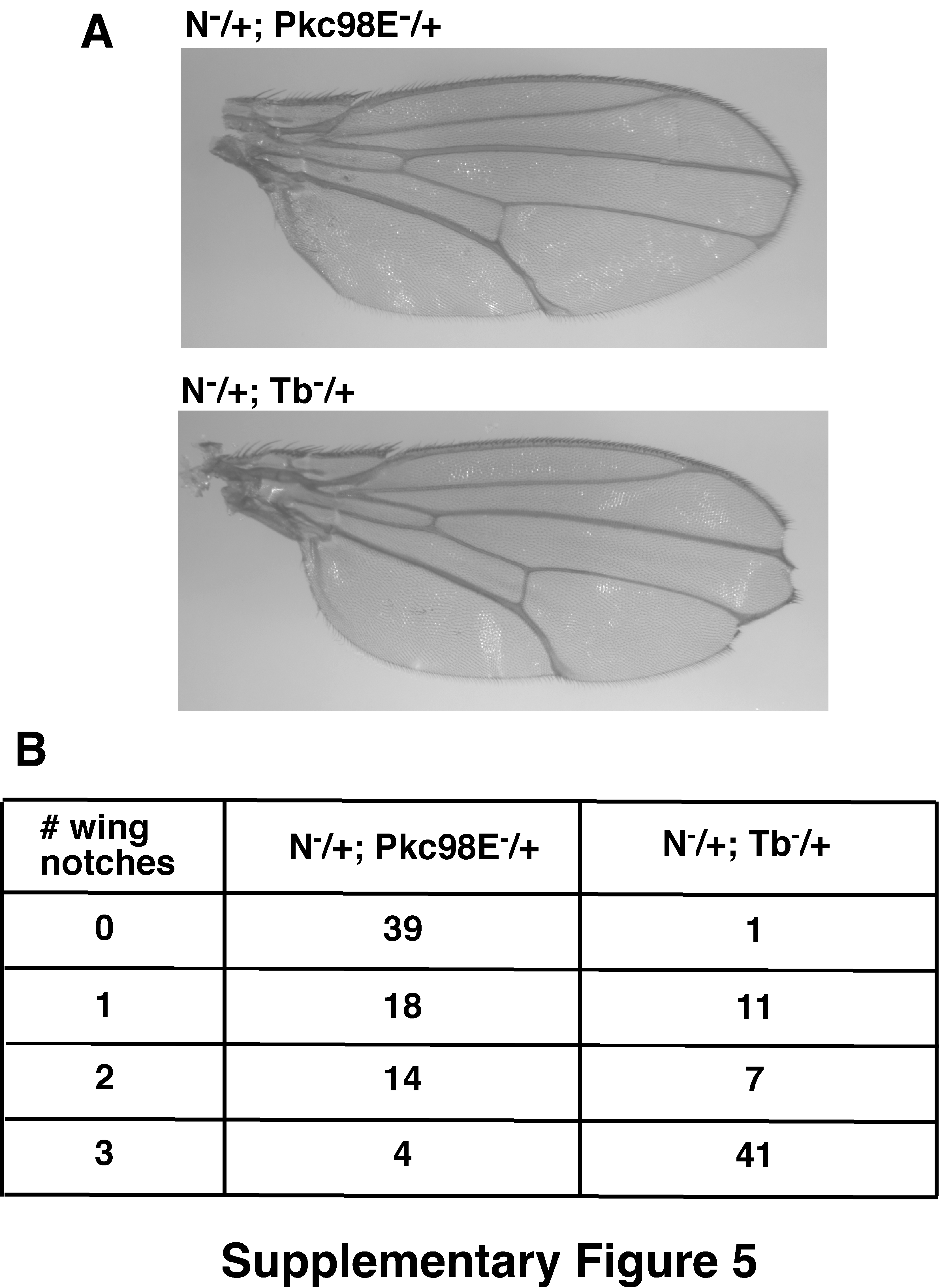

Supplement: Figure S5 — Heterozygosity for Pkc98E suppresses the wing notching phenotype of Notch heterozygous flies. A. Representative wings of flies that are double heterozygotes for Notch and Pkc98E genes and flies that are double heterozygotes for Notch and an unrelated gene Tubby (Tb) on the TM6 balancer chromosome. These flies were siblings from the same cross. B. Table showing the distribution of severity of wing notching in Notch; Pkc98E and Notch; Tubby double heterozygous flies derived from the same cross. (TIF) [file pone.0067789.s005.tif]
